# Nanopatterns of arylene–alkynylene squares on graphite: self-sorting and intercalation

**DOI:** 10.3762/bjoc.15.180

**Published:** 2019-08-02

**Authors:** Tristan J Keller, Joshua Bahr, Kristin Gratzfeld, Nina Schönfelder, Marcin A Majewski, Marcin Stępień, Sigurd Höger, Stefan-S Jester

**Affiliations:** 1 Kekulé-Institut für Organische Chemie und Biochemie, Rheinische Friedrich-Wilhelms-Universität Bonn, Gerhard-Domagk-Str. 1, 53121 Bonn, Germany; 2 Wydział Chemii, Uniwersytet Wrocławski, ul. F. Joliot-Curie 14, 50-383 Wrocław, Poland

**Keywords:** macrocycles, scanning tunneling microscopy, self-assembled monolayers, self-sorting, solid/liquid interface

## Abstract

Supramolecular nanopatterns of arylene–alkynylene squares with side chains of different lengths are investigated by scanning tunneling microscopy at the solid/liquid interface of highly oriented pyrolytic graphite and 1,2,4-trichlorobenzene. Self-sorting leads to the intermolecular interdigitation of alkoxy side chains of identical length. Voids inside and between the squares are occupied by intercalated solvent molecules, which numbers depend on the sizes and shapes of the nanopores. In addition, planar and non-planar coronoid polycyclic aromatic hydrocarbons (i.e., butyloxy-substituted kekulene and octulene derivatives) are found to be able to intercalate into the intramolecular nanopores.

## Introduction

Two-dimensional (2D) nanoporous systems on solid surfaces have gained recent interest in nanosciences and nanotechnology. A precise control of the arrangement/periodicity [1] and pore geometries [2] is crucial for potential applications. Beyond nanofabrication [3–5], as a top-down method, with examples such as focused ion beam milling and subsequent oxygen etching [6], one bottom-up way for the formation of (supported) nanoporous systems is based on the physisorption of molecular species [7]. Non-covalently bound (e.g., hydrogen-bonded) [8–9] nanoporous systems can be formed as a result of intermolecular interactions between directionally bound star-shaped species, such as trimesic acid [10], or 1,3,5-benzenetribenzoic acid [11]. However, they sometimes suffer from a breakdown of an intended packing motif leading to solvent-dependent polymorphism [10]. In addition, guest molecules can act as alien species that affect the morphologies of intermolecular nanopores [12]. Another approach for the formation of nanopores relies on the physisorption of shape-persistent macrocycles [13–14]. While there are examples for the deposition of organic molecules into rigid nanopores from the gas phase [15], the intercalation of organic molecules into nanopores is rather difficult to tailor from scratch, however, with prominent examples [16–17]. Likewise, larger polycyclic aromatic hydrocarbons (PAHs) and nanographenes form robust adsorbate films in a certain size range, and the solubility limit can be overcome by appropriate substitution. We recently investigated self-assembled nanoporous networks of shape-persistent macrocycles in which dithiophene-based corner building blocks connect linear oligo(phenylene–ethynylene–butadiynylene)s (OPEBs) to form molecular polygons with different numbers of sides [18], that are scalable to some extent [19]. The long alkoxy side chains mediate sufficient compound solubility and compound adsorption on highly oriented pyrolytic graphite (HOPG) that acts as a template [20–21], and determine the intermolecular interaction [22–24]. The patterns formed by the polygons, especially the bigons, triangles, squares, and hexagons as well as molecular spoked wheels, are alike if the dithiophene corner pieces are exchanged by other corners, or if the side length is altered [19]. However, in all these cases the alkyl/alkoxy side chains at each side of a specific polygon have the same length. In order to generate more complex superstructures, especially if coadsorbates will be investigated, the question will be addressed, how patterns of polygons containing side chains of different lengths within a given molecule will be formed [25]. In other words, either alkyl/alkoxy chains of the same or different length(s) will interdigitate, which corresponds to narcisstic self-sorting or social self-sorting, respectively [26].

## Results and Discussion

**Figure 1:**
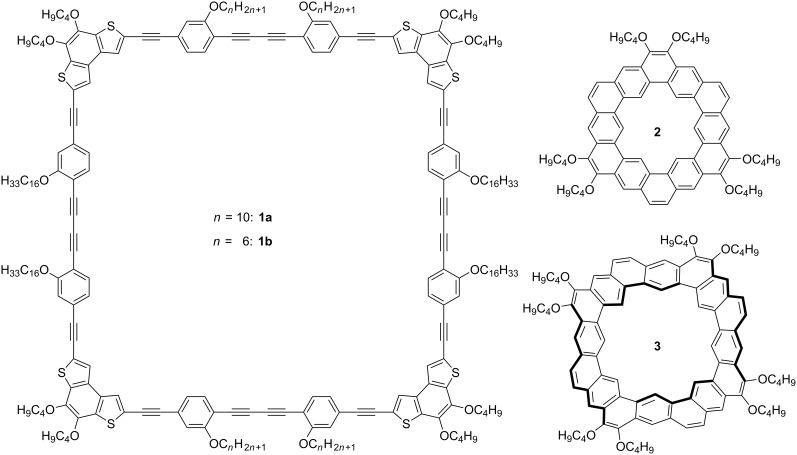
Chemical structures of the molecular squares **1a**/**b**, the kekulene derivative **2**, and octulene derivative **3** [27].

Here, we report on the synthesis and supramolecular nanopatterns of shape-persistent arylene–alkynylene macrocycles **1a**/**b** (Figure 1), and the intercalation of solvent molecules and polycyclic aromatic hydrocarbons (PAHs).

Our way towards nanoporous quadratic templates containing long (OC_16_H_33_) alkoxy side chains on two opposite sides of the square and two shorter (OC_10_H_21_, OC_6_H_13_) side chains on the other sides of the square is based on the Glaser coupling of acetylene-terminated precursor structures. These side chains give macrocycles with reduced symmetry. They cannot be obtained by the cyclization of small building blocks, but require the stepwise formation of more complex precursors, where the information of the chain arrangement in the final molecule structure (and also the 2D nanopattern) is already given in the ring precursor. Therefore, precursor structures were prepared in a stepwise coupling strategy, making use of the trimethylsilyl and the (more polar) (3-cyanopropyl)dimethylsilyl protective groups [28]. Subsequently, **1a**/**b** were prepared by Pd-catalyzed oxidative cyclodimerization of the respective acetylene-terminated precursors under high-dilution conditions [29–30]. The separation of the crude products by recycling gel permeation chromatography (recGPC) yielded the monodisperse compounds **1a**/**b** (see Supporting Information File 1). Details, including the full compound characterization (NMR and MS), are given in Supporting Information File 1.

**Figure 2:**
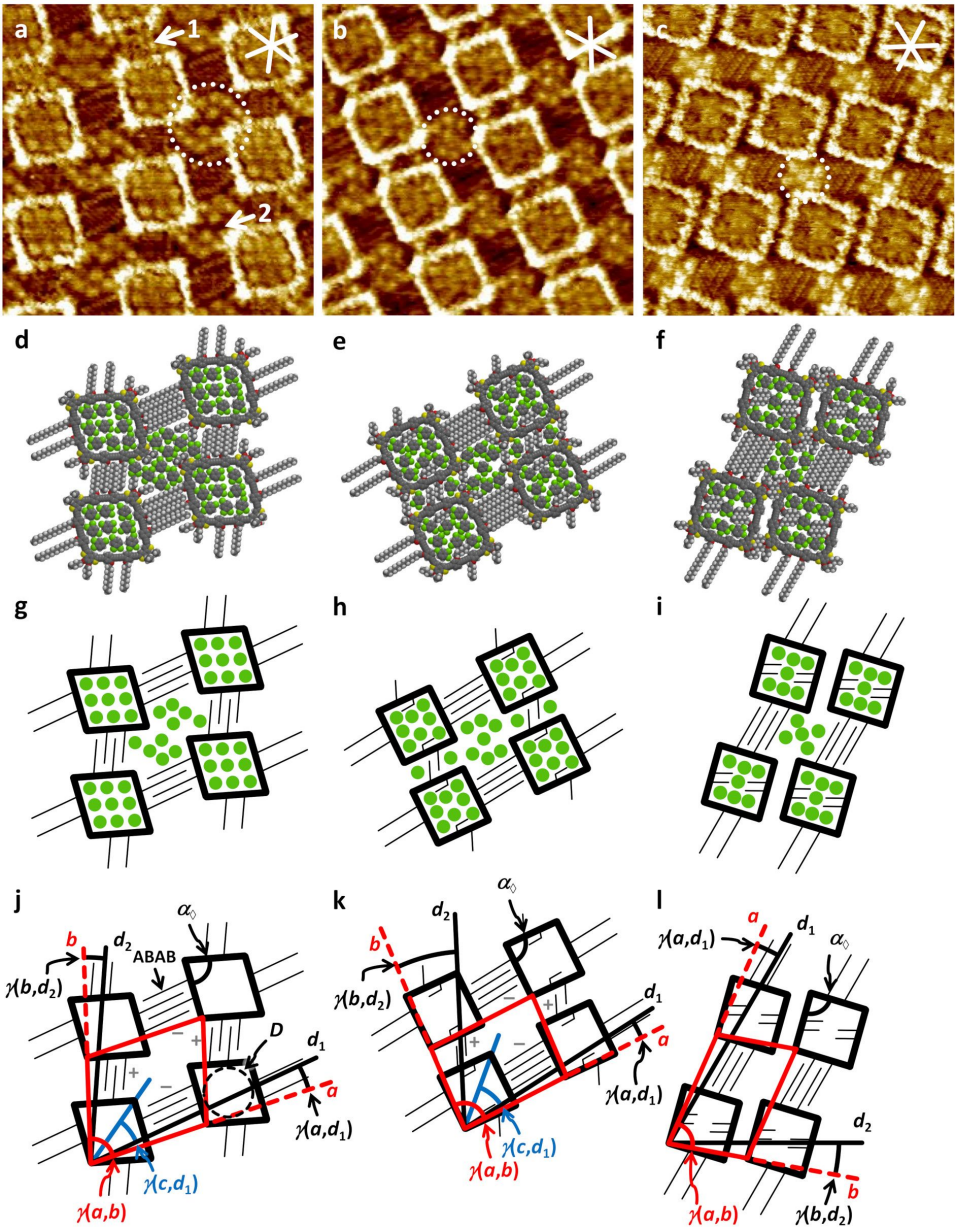
(a)–(c) Scanning tunneling microscopy images, (d)–(f) supramolecular models, and (g)–(l) schematic models of supramolecular nanopatterns of **1a** ((a), (d), (g), (j)), polymorph A of **1b** ((b), (e), (h), (k)), and polymorph B of **1b** ((c), (f), (i), (l)) at the TCB/HOPG interface. Image parameters, unit cells, and additional packing parameters are: (a), (d), (g), (j) **1a**; *c* = 1 × 10^−6^ M, *V*_S_ = −0.8 V, *I*_t_ = 16 pA; *a* = (5.6 ± 0.2) nm, *b* = (4.8 ± 0.2) nm, γ(*a*,*b*) = (74 ± 2)°; γ(*c*,*d_1_*) = (30 ± 4)°; γ(*a*,*d*_1_) = γ(*b*,*d*_2_) = (7 ± 2)°; α_◊_ = (80 ± 4)°; (b), (e), (h), (k) **1b**; *c* = 5 × 10^−7^ M, *V*_S_ = −0.6 V, *I*_t_ = 25 pA; *a* = (5.5 ± 0.2) nm, *b* = (4.2 ± 0.2) nm, γ(*a*,*b*) = (84 ± 2)°; γ(*c*,*d*_1_) = (36 ± 4)°; γ(*a*,*d*_1_) = (4 ± 1)°; γ(*b*,*d*_2_) = (21 ± 2)°; α_◊_ = (87 ± 3)°; (c), (f), (i), (l) **1b**; *c* = 1 × 10^−6^ M, *V*_S_ = −0.4 V, *I*_t_ = 26 pA; *a* = (5.6 ± 0.2) nm, *b* = (4.0 ± 0.2) nm, γ(*a*,*b*) = (74 ± 2)°; γ(*a*,*d*_1_) = (5 ± 1)°; γ(*b*,*d*_2_) = (9 ± 1)°; α_◊_ = (88 ± 4)°. All samples were thermally annealed for 20 s at 80 °C prior to imaging. All image sizes are 15.4 × 15.4 nm^2^. The red lines indicate the unit cells, *a*, *b*, and γ(*a*,*b*). The white and black lines indicate the HOPG main axis directions, *d*_1_, and *d*_2_. Bold and thin black lines in (c) represent backbones and (adsorbed) alkoxy side chains (whereas freely moving side chains, and side chains that point towards the solution phase are omitted). Green dots indicate the positions of intercalated TCB molecules. The diameter of the circle fitted to the rhombic intramolecular nanopore in (j) is *D* ≈ 2.3 nm.

Supramolecular nanopatterns of **1a**/**b** at the solid/liquid interface of HOPG and 1,2,4-trichlorobenzene (TCB) were investigated by scanning tunneling microscopy (STM). Throughout all STM images, surface regions covered by arylene–alkynylene backbones and (intermolecularly interdigitating) alkoxy side chains appear in bright and dark colors, which indicate high and low tunneling currents, respectively [31]. In most of our STM images, we achieve a resolution that allows to detect submolecular features, in some cases down to the resolution of individual CH_2_ units. At a concentration of 1 × 10^−6^ M, **1a** forms a submonolayer surface coverage of an oblique pattern (Figure 2a) with a domain size >50^2^ nm^2^ (with occasional vacancies, see Supporting Information File 1). For this nanopattern, a unit cell of *a* = (5.6 ± 0.2) nm, *b* = (4.8 ± 0.2) nm, γ(*a*,*b*) = (74 ± 2)° is indexed. The long hexadecyloxy (OC_16_H_33_) side chains of each molecule align along one of the HOPG main axis directions (denoted as *d*_1_) and interdigitate with the OC_16_H_33_ side chains of adjacent molecules in an ABAB fashion (see Figure 2j). Likewise, the shorter decyloxy (OC_10_H_21_) side chains of each molecule align along the other HOPG main axis direction *d*_2_, with γ(*d*_1_,*d*_2_) = 60°, and interdigitate with chains of identical length in an ABAB fashion. The supramolecular nanopattern is oriented relative to the HOPG main axis directions with γ(*a*,*d*_1_) = γ(*b*,*d*_2_) = (7 ± 2)°. The backbones are oriented with γ(*c*,*d*_1_) = (30 ± 4)° relative to the HOPG main axis direction *d*_1_. An additional nomenclature describes the orientation of the intermolecularly interdigitating OC_16_H_33_ (and OC_10_H_21_) side chains [32]. The innermost side chain (of each “bundle” of four) as viewed from the center of each intermolecular nanopore) can either be oriented in clockwise or counterclockwise direction, which is indicated by (−) and (+) signs in Figure 2j. Consequently, to the nanopattern of **1a**, OC_16_H_33_ (−); OC_10_H_21_ (+); OC_16_H_33_ (−); OC_10_H_21_ (+) is indexed (Figure 2j). However, a variation of that packing is observed as a packing defect, marked by arrow 1 in Figure 2a, which correlates to OC_16_H_33_ (−); OC_10_H_21_ (−); OC_16_H_33_ (−); OC_10_H_21_ (−) and may have an impact on the respective backbone shape. The (short) butyloxy side chains, that are attached to the corner building blocks to increase the compound’s solubility, do not contribute to the intermolecular packing and remain unresolved by STM. However, dark image regions are observed in the extraannular regions near the dithiophene corner units (e.g., arrow 2 in Figure 2a), as expected for an electrically more insulating surface region. Therefore, we assume that the side chains are either aligned along one of the HOPG main axis directions, or, as an effect of lacking an interaction partner, are mobile to some extent. Moreover, the relative orientation of the hexadecyloxy and decyloxy substituents of the quadrangle sides with γ(*d*_1_,*d*_2_) = 60° leads to a deviation of the (nominally) quadratic backbones towards rhombic-shaped objects with an interior angle of α_◊_= (80 ± 4)°. In addition, some of the high-resolution STM images of **1a** on HOPG (e.g., Figure 2a) show a certain contrast variation in the intra- and intermolecular nanopores (or, the otherwise uncovered regions). More precisely, in all intraannular nanopores that are found in Figure 2a, nine medium bright dots are observed. According to the supramolecular model (Figure 2d), nine solvent (TCB) molecules fit into the intramolecular nanopore, and pack densely in an oblique array of three by three molecules. Additionally, the bright features in the intermolecular regions that are not covered by the interdigitating or mobile alkoxy side chains (cf. dotted circle in Figure 2a) are assigned to ten TCB molecules in a characteristic arrangement of three, two, two, and three molecules (cf. Figure 2d and g).

It has to be noted that solvents like 1-phenyloctane are suitable for STM at the solid/liquid interface, as they do not compete with the adsorbing species of interest [33]. However, alkyl chain-substituted solvent molecules are sometimes resolved as intercalation adducts in STM of self-assembled monolayers. On the other hand, reports on intercalated TCB molecules are rather rare. Two exceptions are the observations of TCB in monolayers of arylene–alkynylene pentagons as well as triphenylene/azobenzene-based molecules on Au(111)) [34–35].

In addition to **1a**, we studied **1b** carrying (long) hexadecyloxy (OC_16_H_33_) and (short) hexyloxy (OC_6_H_13_) side chains, so that the anisotropy of the molecule is slightly increased as compared to **1a**. Compound **1b** assembles (at a concentration of 5 × 10^−7^ M) into an oblique pattern (Figure 2b) with a domain size of >100^2^ nm^2^ (see Supporting Information File 1). For this nanopattern, a unit cell of *a* = (5.5 ± 0.2) nm, *b* = (4.2 ± 0.2) nm, γ(*a*,*b*) = (84 ± 2)° is indexed. Again, the long (hexadecyloxy) and short (hexyloxy) side chains interdigitate with side chains of the same lengths in an ABAB fashion (i.e., narcisstic self-sorting). A side chain interdigitation scheme of OC_16_H_33_ (−); OC_6_H_13_ (+); OC_16_H_33_ (−); OC_6_H_13_ (+) is indexed to the packing observed in Figure 2b (and obviously, the enantiomer has also been observed). The lattice constant *b* for **1b** is reduced by (0.6 ± 0.4) nm as compared to the lattice of **1a**. At first sight, this significant reduction is only attributed to the missing four methylene units in these side chains that define the intermolecular interaction along HOPG main axis direction *d*_2_ (that is about *b*). Such changes are clearly resolvable within the experimental accuracy by calibration of the STM images with the underlying HOPG lattice. A more detailed investigation shows more dramatic changes of the supramolecular nanopattern formed. While the unit cell vector *a* is aligned relative to the HOPG main axis direction *d*_1_ with γ(*a*,*d*_1_) = (4 ± 1)° (and therefore similar as γ(*a*,*d*_1_) observed for **1a**), the shorter lattice vector *b* is aligned with γ(*b*,*d*_2_) = (21 ± 1)°. In addition, the backbones are oriented with γ(*c*,*d*_1_) = (36 ± 4)° relative to the HOPG main axis direction *d*_1_. This is a result of a significant packing change of **1b** as compared to **1a**. Two of the hexyloxy (OC_6_H_13_) side chains of opposing sides of each square are oriented towards the macrocycle interior, and are observed as darker image regions, and the remaining OC_6_H_13_ chains interact intermolecularly. The closer packing of **1b** leads to the interaction of the butyloxy chains of the macrocycle corner units in the monolayer. The changes in the supramolecular lattice are accompanied by subtle changes of the backbone geometries, i.e., an increase of the interior angle α_◊_ to (87 ± 4)° in **1b** (as compared to (80 ± 4)° in **1a**). The changes of the backbone shape together with the partly hexyloxy-filled interior translate into a reduced number of only eight (instead of nine) TCB molecules that pack densely in the intramolecular nanopores. Additionally, only seven TCB molecules (as compared to ten in **1a**) fill the intermolecular voids (cf. white dotted circle in Figure 2b). Both are observed as bright features.

At a higher concentration of **1b** (of 1 × 10^−6^ M) in the supernatant liquid phase, polymorph B is observed, which was indexed with a unit cell of *a* = (5.6 ± 0.2) nm, *b* = (4.0 ± 0.2) nm, γ(*a*,*b*) = (74 ± 2)°. The (long) hexadecyloxy (OC_16_H_33_) side chains are (like in polymorph A) adsorbed along an HOPG main axis direction *d*_1_ with γ(*a*,*d*_1_) = (5 ± 1)°, whereas all (short) hexyloxy side chains point into the macrocycle interior – a result of the unhindered rotation of the *p*-phenylene units prior to the physisorption – or point towards the solution phase, leading to a close contact of the backbones along one direction with a slight offset (most probably due to the butyloxy (OC_4_H_9_) side chains of the macrocycle corners). Consequently, only seven TCB molecules can intercalate in the intramolecular nanopore of the (almost quadratic) backbones (α_◊_ = (87 ± 4)°) and are observed as bright dots (in rows of three, one, and three TCB molecules). In addition, five TCB molecules intercalate into the intermolecular nanopore in a dense packing of two, one, and two molecules (cf. white dotted circle in Figure 2c).

**Figure 3:**
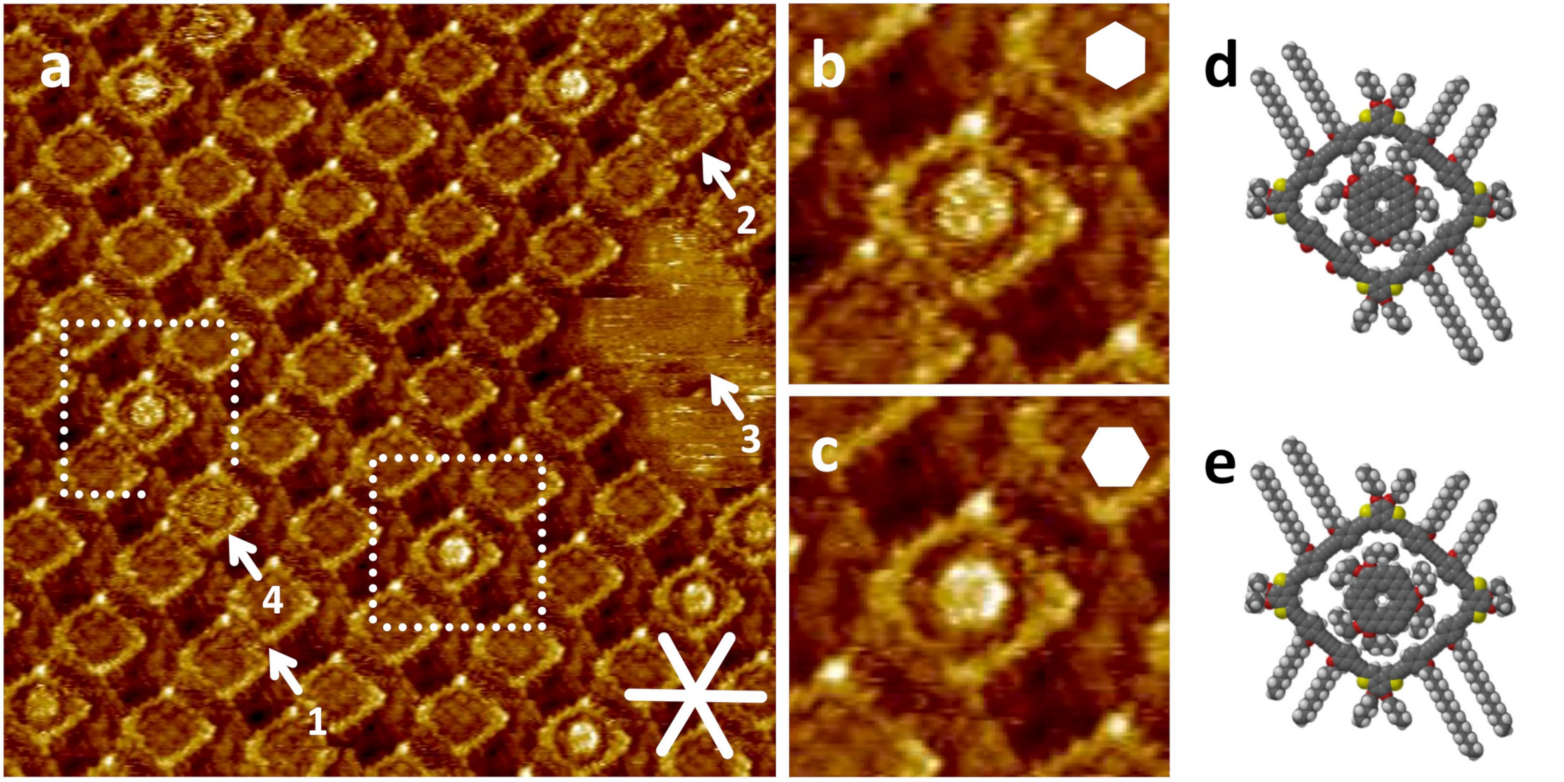
(a) Overview scanning tunneling microscopy image of a nanopattern of **1a** with intermolecularly intercalated **2** at the TCB/HOPG interface, (b)–(c) enlarged cutouts of the marked regions of (a), and (d)–(e) molecular models of the structures observed in (b) and (c). Image parameters and sample preparation: (a) 1 µL *c*(**1a**) = 5 × 10^−7^ M (thermally annealed for 20 s at 80 °C), and 1 µL *c*(**2**) = 1 × 10^−6^ M added at rt; *V*_S_ = −0.7 V, *I*_t_ = 49 pA; 39.5 × 39.5 nm^2^; (b) and (c): 8.7 × 8.7 nm^2^. White lines indicate the HOPG main axis directions.

The observation of intercalated TCB molecules inspired us to investigate, whether the nanopores can also host larger species, such as planar and non-planar circulenes, and which preparation conditions are required for this purpose. Kekulene derivative **2** carries six (solubility-enhancing) butyloxy side chains in pairwise arrangements, and therefore the *D*_6_*_h_* symmetry of the (oblate) backbones is reduced to *D*_3_*_h_* [27]. At the solid/liquid interface of solutions of **2** in a concentration range of 10^−3^ M to 10^−5^ M in TCB on HOPG, self-assembled monolayers of **2** are found (see Supporting Information File 1). We studied whether **2** can adsorb into the nanopores formed by **1a**. Therefore, first a nanopattern of **1a** was (self-)assembled from a 5 × 10^−7^ M solution of **1a** to HOPG (at an elevated temperature, 80 °C), and (after cooling of the sample to rt) a 1 × 10^−6^ M solution of **2** in TCB was added. By STM (Figure 3a), a monolayer of **1a** (polymorph A) is observed (and expected in that concentration range, cf. Figure 2a). However, some rows of more densely packed molecules (polymorph B) are observed, where two (arrow 1 in Figure 3a) or all four (arrow 2 in Figure 3a) decyloxy side chains are oriented towards the intraannular region. This is consistent with the spatial requirements of **2**, which compete with the adsorption of **1a**, and lead to blurred regions as marked by arrow 3 in Figure 3a. In addition, nine of the 59 intraannular regions (i.e., 15%) in Figure 3a host a molecule of **2** (up to 17%, see Supporting Information File 1). Eight species of **2** (in Figure 3a) appear as bright features (with a slight central depression), whereas arrow 4 in Figure 3a marks a blurry molecule of **2**. We expect that the rotation of the brightly appearing molecules of **2** (in the nanopores) is hindered by coadsorbed TCB molecules that fill the remaining intraannular region between **2** and the macrocycle rim (cf. Figure 3d and e, however TCB not shown for clarity). Enlarged areas of Figure 3a (shown in Figure 3b and c) show two template macrocycles **1a** (of similar orientation), each of which is filled with one molecule of **2** that appears as a bright hexagon – however, with different orientations. The hexagons of the nanographene **2** in Figure 3c and e have the same orientation as the hexagons in the underlying graphene layer, and therefore the same orientation as proposed in the self-assembled monolayer of pure **2** (see Supporting Information File 1), and other large PAHs on HOPG [36]. However, **2** in Figure 3b and d is rotated by 30°. This is rather unexpected for such small molecular species. Moreover, while misorientations are commonly observed (e.g., in turbostratic graphite) [37], such a controlled rotation of (nano-)graphene(s) is rather difficult to achieve [38], but may exhibit unexpected electronic properties [39]. In addition, the behavior of molecules in nanoporous systems can be different as compared to the unhindered graphene lattice [40]. We can only speculate on the roles of the *D*_3_*_h_* symmetry of **2** vs. the *D*_2_*_h_* symmetry of the nanopore of **1a** (and its relative orientation to the graphite lattice). An observation of **2** in a more dense arrangement of **1a** (e.g., as seen in Figure 3b) is found, indicating that the decyloxy side chains of the southwest side must be oriented towards the solution phase.

**Figure 4:**
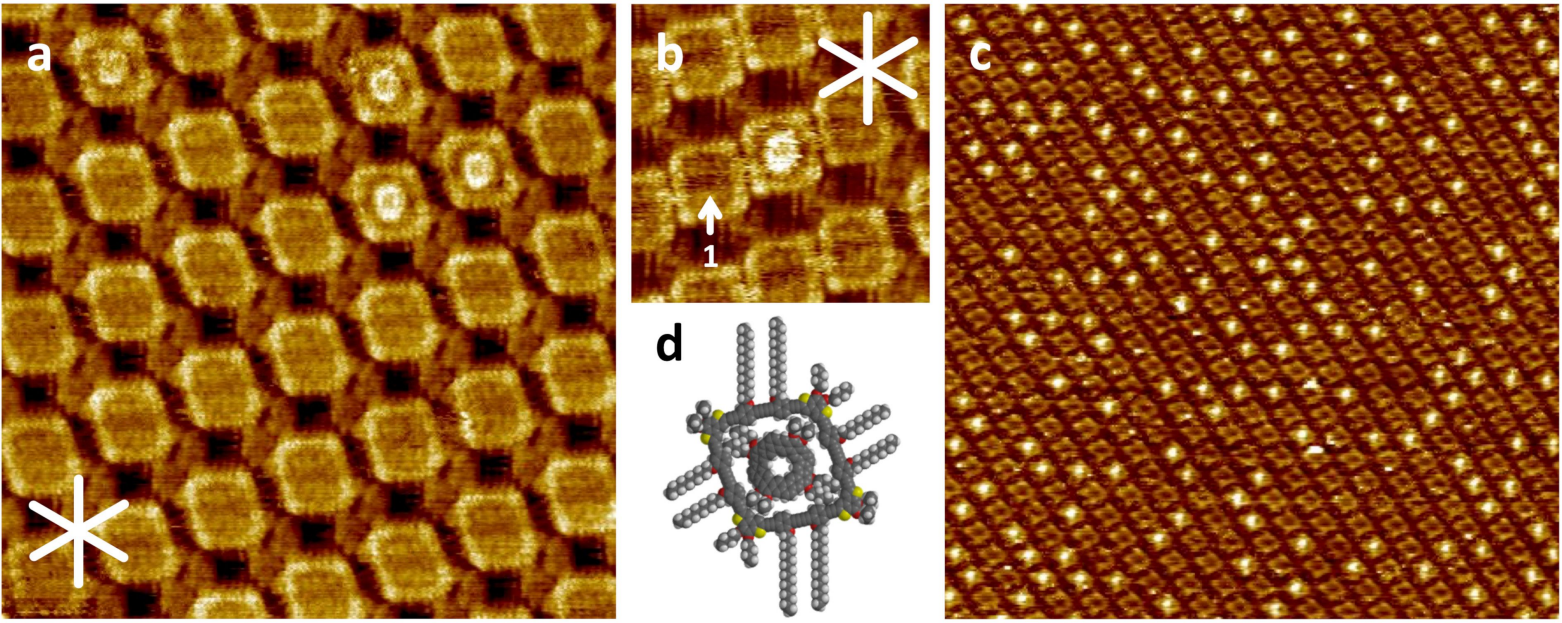
(a–c) Scanning tunneling microscopy images of a nanopattern of **1a** with intermolecularly intercalated **3** at the TCB/HOPG interface. Image parameters and sample preparation: (a) 1 µL *c*(**1a**) = 5 × 10^–7^ M (thermally annealed for 20 s at 80 °C), and 1 µL *c*(**2**) = 1 × 10^−4^ M added at rt; *V*_S_ = −0.5 V, *I*_t_ = 24 pA; 31.2 × 31.2 nm^2^; (b) 1 µL *c*(**1a**) = 5 × 10^−7^ M (thermally annealed for 20 s at 80 °C), and 1 µL *c*(**2**) = 1 × 10^−4^ M added at rt; *V*_S_ = −0.8 V, *I*_t_ = 34 pA; 14.8 × 14.8 nm^2^; (c) 1 µL *c*(**1a**) = 5 × 10^−7^ M (thermally annealed for 20 s at 80 °C), and 1 µL *c*(**2**) = 1 × 10^−3^ M added at rt; *V*_S_ = −0.7 V, *I*_t_ = 44 pA; 158 × 158 nm^2^ and (d) proposed intercalation model. White lines indicate the HOPG main axis directions.

Next, we studied the pore-filling by the non-planar octulene derivative **3** [27]. (Pure) **3** does not form 2D-crystalline monolayers at the TCB/HOPG (and also the 1-octanoic acid/HOPG) interface at concentrations as low as 10^−3^ M or lower (see Supporting Information File 1). Anyhow, the above results motivated us to investigate whether **1a** stabilizes the assembly by intercalation of the non-planar species **3**. Therefore, we prepared (by the procedure described above) a nanopattern of **1a** (5 × 10^−7^ M), and added a 10^−4^ M solution of **3** in TCB. STM (Figure 4a) shows – again – bright features in the macrocyclic template of **1a**. Again, a slight central depression is visible in the STM image which can attributed to the empty center region of **3**. As shown in the proposed supramolecular model in Figure 4d, the interior of the intramolecular pore is able to incorporate **3** despite its larger diameter compared to **2**. At the above concentrations, roughly 3% of the macrocycles **1a** are filled with the octulene derivative (see Supporting Information File 1). At these conditions, **1a** can also be found in its denser polymorph (see Figure S2 in Supporting Information File 1). Overview STM images (see Supporting Information File 1) of both polymorphs (A and B) show a higher affinity of **3** to the intraannular regions of polymorph A (allowing unhindered access to the intraannular space, cf. Figure 4a) as compared to polymorph B (where the side chains may point towards the macrocycle interior, cf. arrow 1 in Figure 4b). This indicates, that the coadsorption of **3** is hindered when it has to compete with the alkyl chains of the macrocycle, and overview STM images comprising both polymorphs are shown in Supporting Information File 1, Figure S9a and b. Therefore, the PAH coadsorption is a monitor for the alkoxy chain orientation inside the macrocyclic interior (whereas they do not point towards the solution phase here). By adding a solution of **3** at a concentration of 1 × 10^−3^ M to a freshly prepared template surface of **1a** (as described above), the overall degree of occupation increased to about 30% in Figure 4c (for full-size and detail images, see Supporting Information File 1). Moreover, when applying a 10^−2^ M solution of **3** to a freshly prepared template of **1a**, an increased degree of occupation up to 66% is observed (see Supporting Information File 1).

The results show, that 2D nanopatterns of molecular squares on graphite behave as crystalline sponges, a phenomenon that has been previously described in 3D [41]. It has to be mentioned that with concentrations of 10^−3^ M and below, **3** does not form a PAH monolayer (or islands/a submonolayer coverage). Only when a higher (10^−2^ M) concentrated solution of **3** is applied to the HOPG surface without the underlying macrocycle template, a self-assembled monolayer of **3** is observed (see Supporting Information File 1).

## Conclusion

Arylene–alkynylenes that carry alkoxy side chains form, after adsorption to graphite, a nanopattern with intra- and intermolecular nanopores. Clusters of otherwise unhinderedly mobile 1,2,4-trichlorobenzene solvent molecules are found in these nanopores. Subtle changes of the pore geometries and/or side chains pointing towards the pore interior translate into varying numbers of intercalated solvent molecules in the intraannular regions. In addition, the intermolecular pores are scalable by the lengths of the alkoxy side chains in a certain range. We showed that the nanopores host alkoxy-substituted kekulene and octulene derivatives.

## Supporting Information

File 1 Synthetic details of **1a**/**b**, characterization, and additional STM images.
