# Supplementary material for: Nanopatterns of arylene–alkynylene squares on graphite: self-sorting and intercalation
Source: Beilstein J Org Chem. 2019 Aug 2;15:1848–55. doi: 10.3762/bjoc.15.180 (PMC6693369; doi:10.3762/bjoc.15.180)
Supplement: File 1 — Synthetic details of 1a/b, characterization, and additional STM images. [file Beilstein_J_Org_Chem-15-1848-s001.pdf]

BEILSTEIN JOURNAL OF ORGANIC CHEMISTRY

Supporting Information

for

Nanopatterns of arylene–alkynylene squares on graphite: self-sorting and intercalation

Tristan J. Keller, Joshua Bahr, Kristin Gratzfeld, Nina Schönfelder, Marcin A. Majewski, Marcin Stępień, Sigurd Höger and Stefan-S. Jester

Beilstein J. Org. Chem. **2019,** *15,* 1848–1855. doi:10.3762/bjoc.15.180

Synthetic details of 1a/b, characterization, and additional STM images

License and Terms: This is a supporting information file under the terms of the Creative Commons Attribution License (http://creativecommons.org/licenses/by/4.0). Please note that the reuse, redistribution and reproduction in particular requires that the authors and source are credited.
The license is subject to the *Beilstein Journal of Organic Chemistry* terms and conditions: (https://www.beilstein-journals.org/bjoc)

Table of contents

1 General information S2

1.1 Materials and equipment S2

1.2 GPC experiment S2

1.3 STM experiment S2

2 Additional scanning tunneling microscopy images S4

2.1 Additional STM images of **1a** S4

2.2 Additional STM images of **1b** S6

2.3 STM images of **2** S9

2.4 STM images of **3** S10

2.5 Additional STM images of **1a** and **2** S11

2.6 Additional STM images of **1a** and **3** S13

3 Synthesis S15

3.1 Synthetic strategy S15

3.2 Synthesis of the condensed dithiophene corner building block **5** S18

3.3 Synthesis of the rigid rod segments **9a/b** S19

3.4 Synthesis of the macrocycles **1a/b** S22

1. General information

1.1 Materials and equipment

Reagents were purchased at reagent grade from commercial sources and used without further purification. All air-sensitive reactions were carried out using standard Schlenk techniques under argon. Reaction solvents (THF, piperidine, dichloromethane, pyridine, triethylamine, toluene) were dried, distilled, and stored under argon according to standard methods. Workup solvents were either used in "p.a." quality or purified by distillation (dichloromethane (DCM), cyclohexane (CH)). Prior to characterization and further processing, all solids and oils were dried at room temperature (rt) under vacuum. ^{1}H and ^{13}C NMR spectra were recorded on a Bruker Avance I 300 MHz, Bruker Avance I 400 MHz, Bruker Avance I 500 MHz, and Bruker Avance III HD 500 MHz Prodigy (300.1, 400.1, and 500.1 MHz for ^{1}H and 75.5, 100.6, and 125.8 MHz for ^{13}C). Chemical shifts are given in parts per million (ppm) referenced to residual ^{1}H or ^{13}C signals in deuterated solvents. All NMR spectra were recorded at rt unless otherwise described. Mass spectra were measured on a Thermo Finnigan ThermoQuest sector field mass spectrometer MAT 95 XL (EI-MS), a Thermo Finnigan sector field mass spectrometer MAT 90 (EIMS), a Bruker Daltonics micrOTOF-Q (ESIMS, APCI), a Thermo Fisher Scientific Orbitrap XL (ESIMS), and a Bruker Daltonics autoflex TOF/TOF (MALDIMS; matrix material: DCTB, no salts added). *m*/*z* peaks smaller than 10% (compared to the basis peak) are not reported. Thin layer chromatography was conducted on silica gel-coated aluminum plates (Macherey-Nagel, Alugram SIL G/UV254, 0.25 mm coating with fluorescence indicator). Silica gel Kieselgel 60 (Merck, 0.040–0.063 mm) was used as the stationary phase for column chromatography.

1.2 GPC experiment

Gel permeation chromatography (GPC) was performed in THF (HPLC grade, stabilized with 2.5 ppm BHT) at rt. GPC analyses were run on an Agilent Technologies system at a flow rate of 1 mL/min using an IsoPump G1310 A, a diode array UV detector (G1315B), and PSS columns (Polymer Standards Service, Mainz, Germany; 10^2, 10^3, 10^5 and 10^6 Å, 5 µ, 8 × 300 mm). All molecular weights were determined versus PS calibration (PS standards from PSS, Mainz, Germany).

For the preparative separation, a Shimadzu Recycling GPC system, equipped with an LC-20 AD pump, an SPD-20 A UV detector and a set of three preparative columns from PSS (either SDV 10^3 Å, 5 µ, 20 × 300 mm or SDV preparative linear S, 5 µ, 20 × 300 mm) with precolumn (SDV, 5 µ, 20 × 50 mm) was employed. The system operated at a flow rate of 5 mL/min, THF, 35 °C.

1.3 STM experiment

Scanning tunneling microscopy (STM) is performed under ambient conditions (rt) at the solution/solid interface, using 1,2,4-trichlorobenzene (TCB) as solvent and highly oriented pyrolytic graphite (HOPG) as substrate. In a typical experiment, 0.2–3 µL of a 5×10^{-7} to 1×10^{-4} M solution of the compound of interest (**1a**/**b**, **2**, or **3**) is dropped onto a freshly cleaved HOPG substrate at elevated temperature (e.g., 80 °C, as indicated in the figure caption), kept at this temperature for a short time (e.g., 20 s), and allowed to cool to rt before the STM measurements are performed with the tip immersed into the solution. Intercalation experiments were performed by preparing the macrocycle template (by applying a solution of **1a**/**b** at an elevated temperature of e.g., 80 °C), allowing the sample to cool to rt, and subsequently adding the same volume of a 1×10^{-6} to 1×10^{-5}

M solution of **2** (or **3**, in TCB) to the sample. Therefore, the absolute concentrations decrease (due to doubling the volume) by a factor of 2. (Sample) bias voltages between −0.4 V and −1.6 V and tunneling current set points in the range of 5 pA to 100 pA are typically used to image the supramolecular adlayers, and exact values are given in the figure captions. The experimental setup consists of an Agilent 5500 scanning probe microscope that is placed on a Halcyonics actively isolated microscopy workstation. It is acoustically shielded with a home-built box. Scissors cut Pt/Ir (80:20) tips were used and further modified after approach by applying short voltage pulses until the desired resolution was achieved. HOPG was obtained from TipsNano (via Anfatec) in ZYB-SS quality. All STM images (unless otherwise noted) were calibrated by subsequent immediate acquisition of an additional image (of same size) at reduced bias voltage (using the same scan rate), in which the atomic lattice of the HOPG surface is observed which is used as a calibration grid. Data processing, also for image calibration, was performed using the SPIP 5 (Image Metrology) software package. (Supra-) molecular modeling was performed using Wavefunction Spartan ’10, ’16, and ‘18.

2. Additional scanning tunneling microscopy images

2.1 Additional STM images of 1a

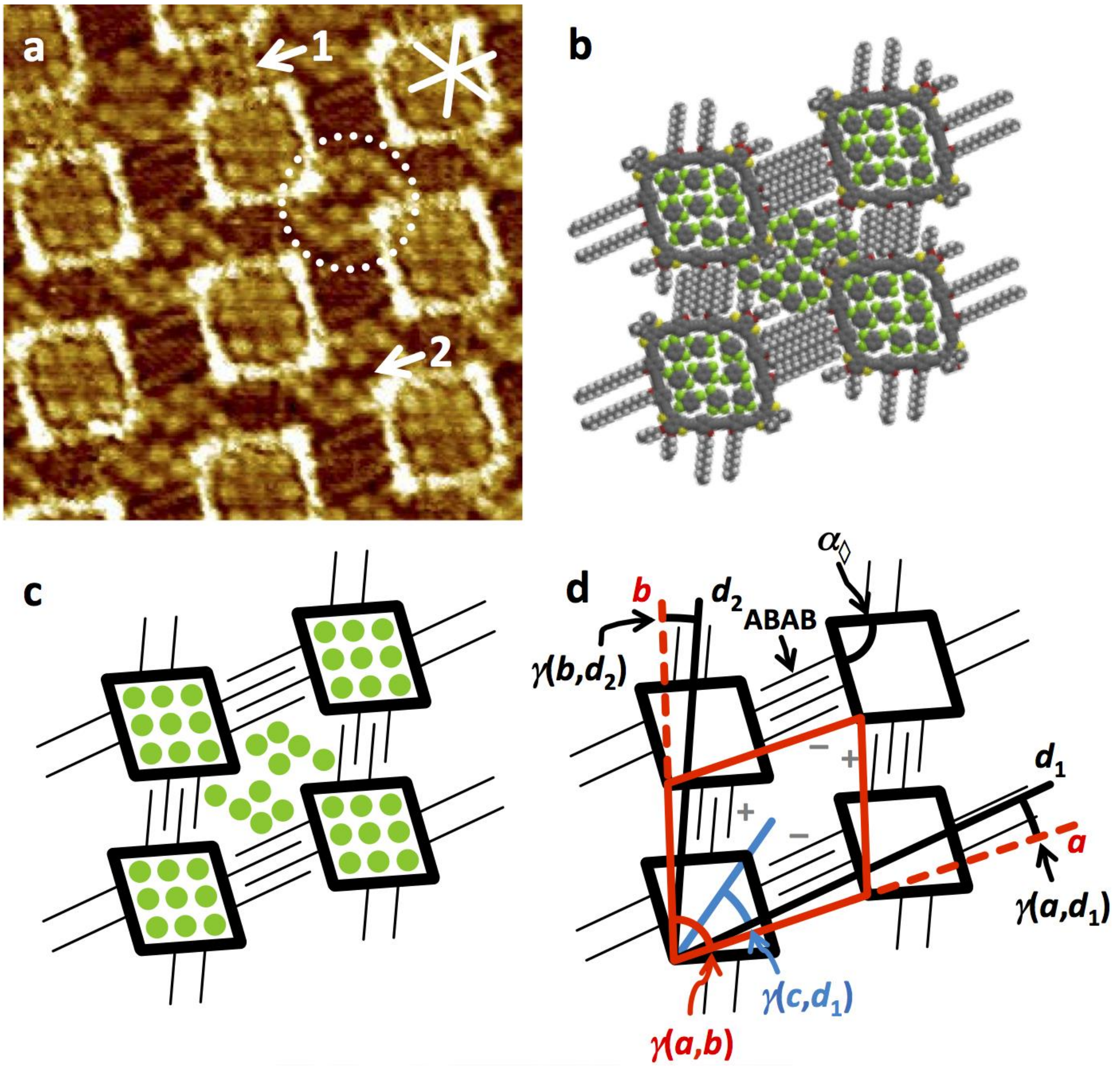

Figure S1: (a) Scanning tunneling microscopy image, (b) supramolecular model, and (c)–(d) schematic models of supramolecular nanopattern of **1a** at the TCB/HOPG interface. Image parameters, unit cells, and additional packing parameters are: $c = 1 \times 10^{-6}$ M, $V_S = -0.8$ V, $I_t = 16$ pA; $a = (5.6 \pm 0.2)$ nm, $b = (4.8 \pm 0.2)$ nm, $\gamma(a,b) = (74 \pm 2)°$; $\gamma(c,d_1) = (30 \pm 4)°$; $\gamma(a,d_1) = \gamma(b,d_2) = (7 \pm 2)°$; $\alpha_\Diamond = (80 \pm 4)°$, image size 15.4 × 15.4 nm^2. Sample thermally annealed for 20 s at 80 °C. The red lines indicate the unit cells, *a*, *b*, and $\gamma(a,b)$. The white and black lines indicate the HOPG main axis directions, d_1, and d_2. Bold and thin black lines in (c)–(d) represent backbones and (adsorbed) alkoxy side chains (whereas freely moving side chains, and side chains that point towards the solution phase are omitted). Green dots indicate the positions of intercalated TCB molecules while blue lines indicate the overall orientation of the backbones.

1a forms large scale self-assembled monolayers at the liquid solid interface of TCB and HOPG of beyond 100×100 nm^2 as shown in Figure S2. The white dotted line in Figure S2a shows a rather straight domain boundary between the self-assembled monolayer of **1a** a wide polymorph A and uncovered/dynamically covered areas. As the longer alkoxy side chains provide a more robust packing with respect to the shorter alkoxy side chains, the domain boundary follows the more stable hexadecyloxy side chain direction. Arrow 1 indicates a packing defect where 3 molecular squares are missing. In Figure S2b, both the wide polymorph A and a dense polymorph B analogous to that in Figure 2c (main text) are present. A typical domain boundary between polymorph A and B is indicated by a white dotted line. Along this direction parallel to the hexadecyloxy side chains, polymorph A and B are commensurable. Packing defects occur along the less robust packing motif caused by the shorter alkoxy side chains shown by arrows 2 and 2'. Again, rather straight domain boundaries are observed along the more robust packing direction as indicated by arrow 3. The uncovered region between those boundaries occurs due to opposing domains being slightly shifted with respect to each other; this offset is clearly visible at the line defect highlighted by arrow 4.

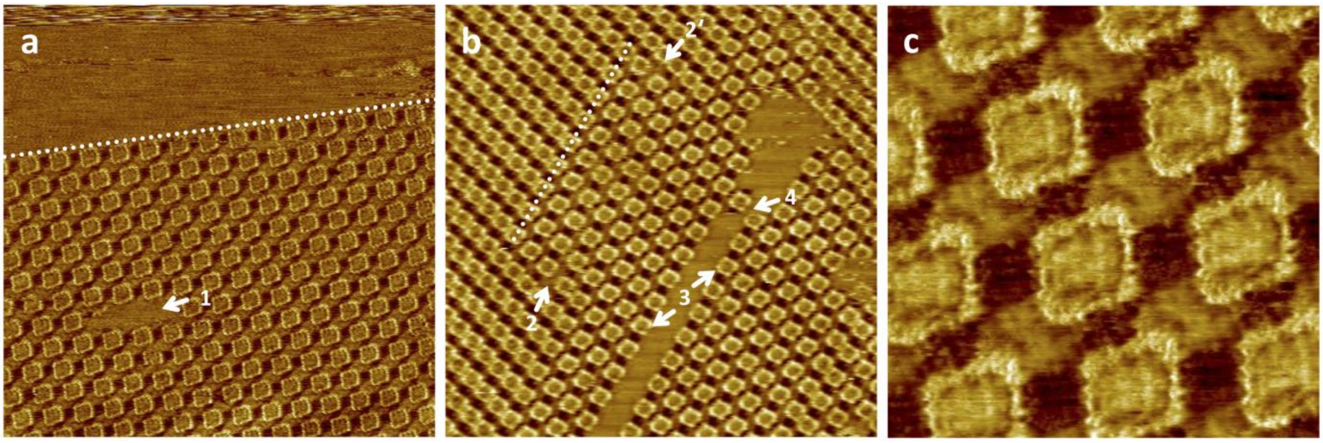

Figure S2: (a)–(c) Self-assembled monolayers of **1a** at the TCB/HOPG interface. (a)–(c) Scanning tunneling microscopy images; image parameters: a: 100×100 nm^2 (internal scanner calibration); $c = 1 \times 10^{-6}$ M; $V_S = -0.71$ V; $I_t = 16$ pA; b: 100×100 nm^2 (internal scanner calibration); $c = 1 \times 10^{-6}$ M; $V_S = -0.6$ V; $I_t = 36$ pA; c: $c = 1 \times 10^{-6}$ M; $V_S = -0.7$ V; $I_t = 13$ pA. Sample thermally annealed for 20 s at 80 °C.

2.2 Additional STM images of 1b

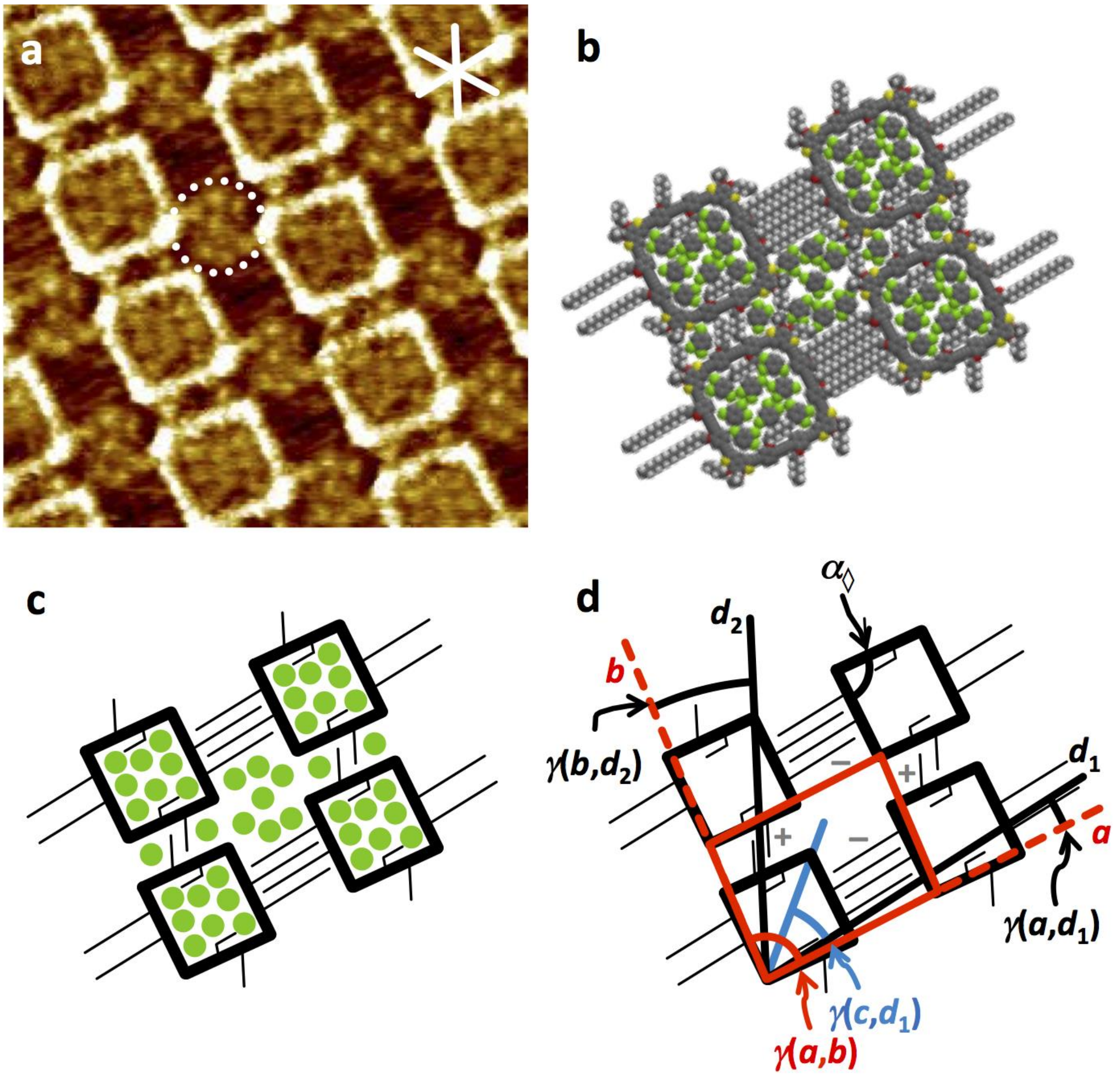

Figure S3: (a) Scanning tunneling microscopy image, (b) supramolecular model, and (c)–(d) schematic models of supramolecular nanopattern of **1b** (polymorph A) at the TCB/HOPG interface. Image parameters, unit cells, and additional packing parameters are: $c = 5 \times 10^{-7}$ M, $V_S = -0.6$ V, $I_t = 25$ pA; $a = (5.5 \pm 0.2)$ nm, $b = (4.2 \pm 0.2)$ nm, $\gamma(a,b) = (84 \pm 2)°$; $\gamma(c,d_1) = (36 \pm 4)°$; $\gamma(a,d_1) = (4 \pm 1)°$; $\gamma(\mathrm{b},d_2) = (21 \pm 2)°$; $\alpha_\Diamond = (87 \pm 3)°$; image size 15.4 × 15.4 nm^2. Sample thermally annealed for 20 s at 80 °C. The red lines indicate the unit cells, *a*, *b*, and $\gamma(a,b)$. The white and black lines indicate the HOPG main axis directions, d_1, and d_2. Bold and thin black lines in (c)-(d) represent backbones and (adsorbed) alkoxy side chains (whereas freely moving side chains, and side chains that point towards the solution phase are omitted). Green dots indicate the positions of intercalated TCB molecules while blue lines indicate the overall orientation of the backbones.

1b forms self-assembled monolayers at the solid liquid interface of HOPG and TCB. Large domain sizes beyond 100×100 nm^2 are observed.

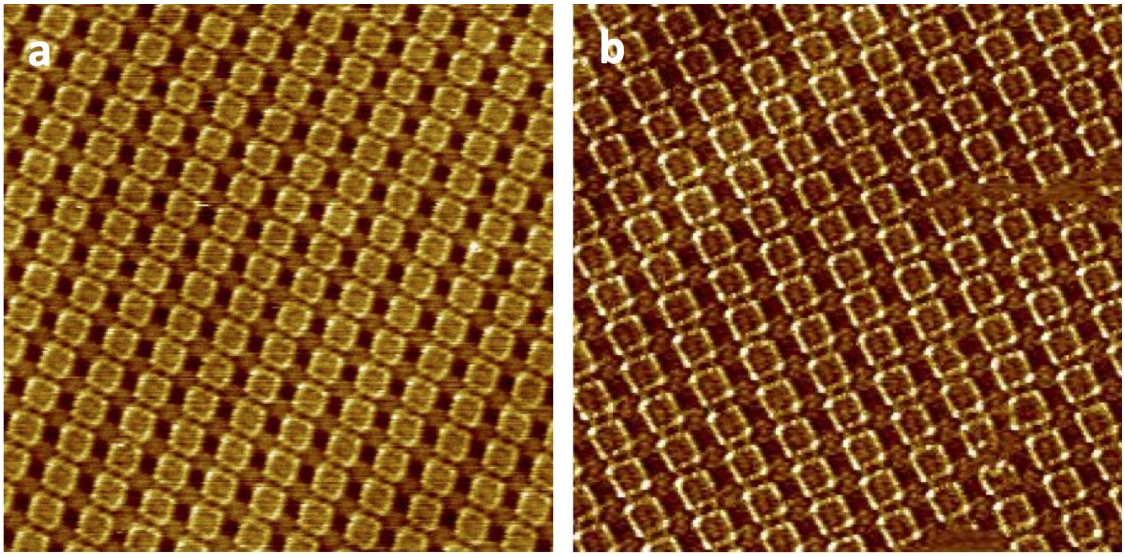

Figure S4: (a)–(b) Self-assembled monolayer of **1b** at the TCB/HOPG interface. (a)–(b) Scanning tunneling microscopy images; image parameters: a: 66.9×66.9 nm^2 (internal scanner calibration); $c = 3 \times 10^{-7}$ M; $V_S = -0.81$ V; $I_t = 46$ pA; b: 60.0×60.0 nm^2; $c = 5 \times 10^{-7}$ M; $V_S = -0.8$ V; $I_t = 35$ pA. Sample thermally annealed for 20 s at 80 °C.

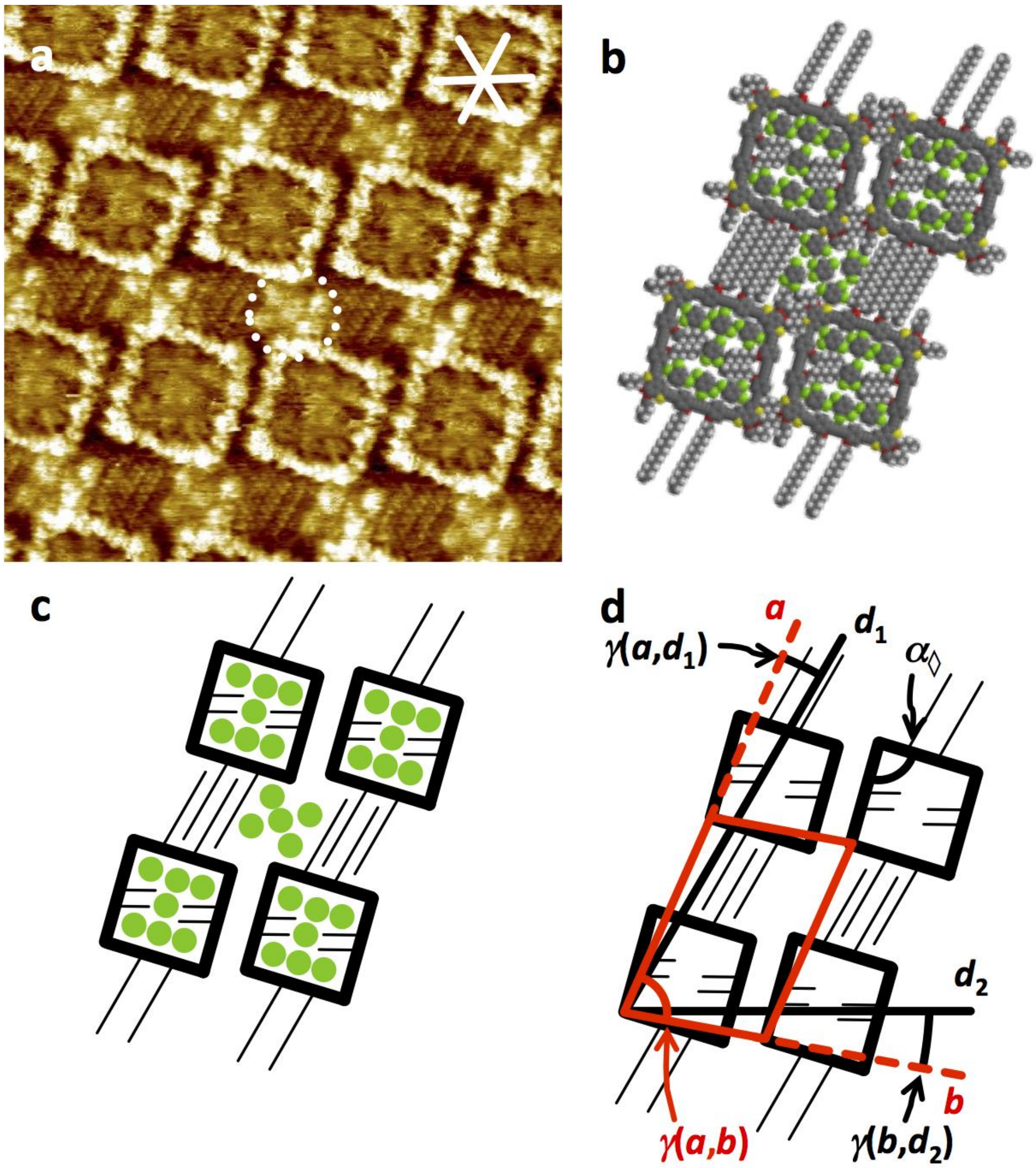

Figure S5: (a) Scanning tunneling microscopy image, (b) supramolecular model, and (c)–(d) schematic models of supramolecular nanopattern of **1b** (polymorph A) at the TCB/HOPG interface. Image parameters, unit cells, and additional packing parameters are: $c = 1 \times 10^{-6}$ M, V_S = −0.4 V, I_t = 26 pA; a = (5.6 ± 0.2) nm, b = (4.0 ± 0.2) nm, $\gamma(a,b)$ = (74 ± 2)°; $\gamma(c,d_1)$ = (36 ± 4)°; $\gamma(a,d_1)$ = (5 ± 1)°; $\gamma(b,d_2)$ = (9 ± 2)°; $\alpha_\Diamond$ = (88 ± 3)°; image size 15.4 × 15.4 nm^2. Sample thermally annealed for 20 s at 80 °C. The red lines indicate the unit cells, a, b, and $\gamma(a,b)$. The white and black lines indicate the HOPG main axis directions, d_1, and d_2. Bold and thin black lines in (c)–(d) represent backbones and (adsorbed) alkoxy side chains (whereas freely moving side chains, and side chains that point towards the solution phase are omitted). Green dots indicate the positions of intercalated TCB molecules.

2.3 STM images of 2

2 self-assembles at the solid/liquid interface of 1,2,4-trichlorobenzene (TCB) and highly oriented pyrolytic graphite (HOPG) into a monolayer forming ordered domains beyond sizes of 100 × 100 nm^2. To the two-dimensional (2D) crystal, a unit cell of $a = b = (2.1 \pm 0.1)$ nm, $\gamma(a,b) = (60 \pm 2)°$ is indexed, and a and b are oriented along the substrate main axis directions. The butyloxy side chains of each molecule cover the regions between the kekulene backbones and determine the intramolecular spacing. The exact rotation of **2** relative to the HOPG lattice is not submolecularly resolved. However, we assume that the orientation of the aromatic rings aligns with the orientation of the underlying graphite substrate.

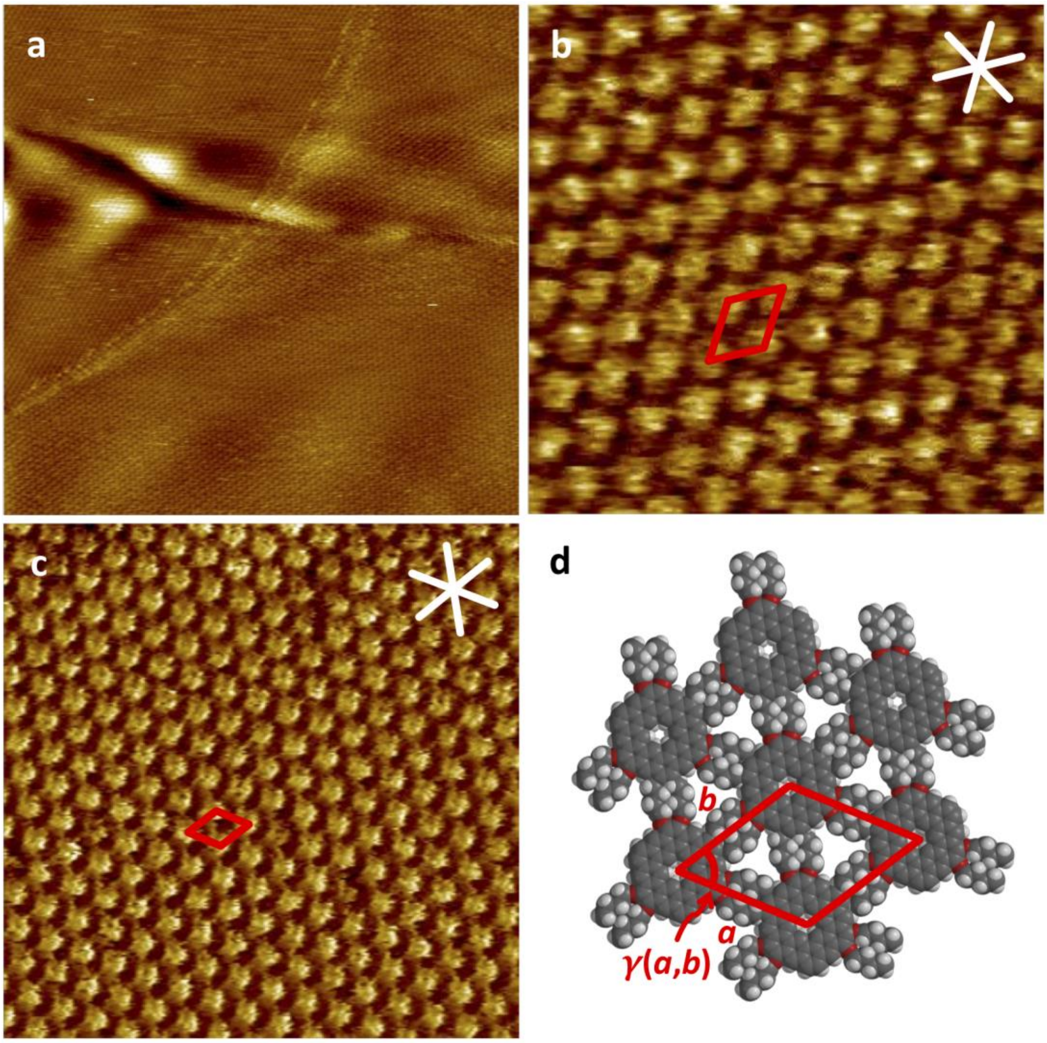

Figure S6: Self-assembled monolayer of **2** at the TCB/HOPG interface. (a)–(c) Scanning tunneling microscopy images; image parameters: a: 190 × 190 nm^2 (internal scanner calibration); $c = 10^{-3}$ M; $V_S = -1.0$ V; $I_t = 30$ pA; b: 20.0 × 20.0 nm^2; $c = 10^{-3}$ M; $V_S = -0.6$ V; $I_t = 15$ pA; c: 30.0 × 30.0 nm^2; $c = 10^{-4}$ M; $V_S = -1.2$ V; $I_t = 30$ pA; all samples thermally annealed for 20 s at 80 °C; unit cell: $a = b = (2.1 \pm 0.1)$ nm, $\gamma(a,b) = (60 \pm 2)°$; (d) proposed supramolecular model. Red and white lines indicate unit cell and HOPG main axis directions, respectively.

2.4 STM images of 3

3 does not form self-assembled monolayers at the solid liquid interface between TCB and HOPG at concentrations up to 10^{-3} M. Figure S7a and b show typical STM images obtained when applying a droplet of a solution of **3** in TCB onto a freshly cleaved HOPG surface. Natural graphite step edges in Figure S7a and b prove the tip quality during the measurement. However, at an increased concentration of 10^{-2} M, a self-assembled monolayer is observed to which a unit cell of $a = b = (2.0 \pm 0.1)$ nm, $\gamma(a,b) = (60 \pm 2)°$ is indexed.

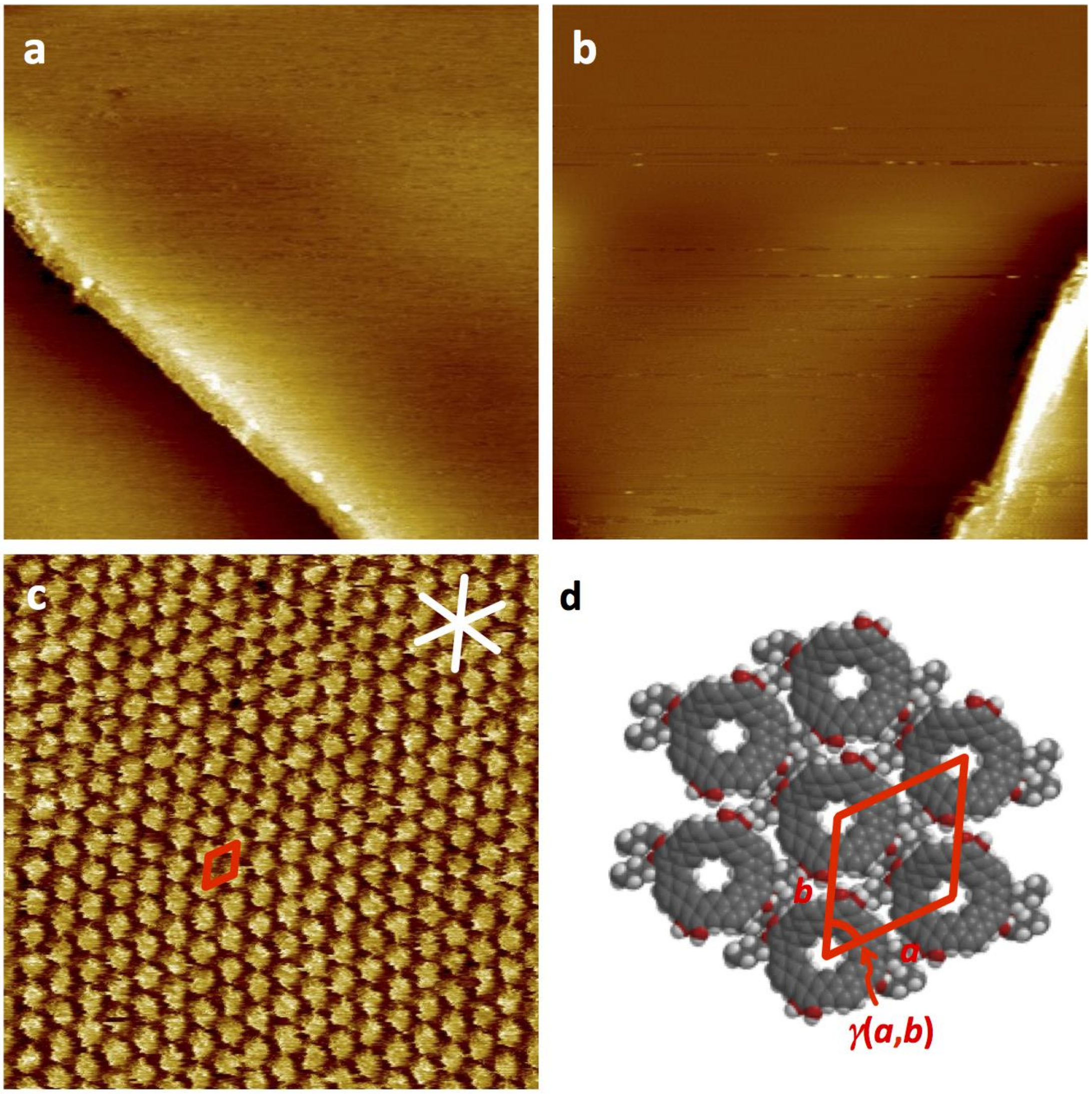

Figure S7: (a)–(c) Scanning tunneling microscopy images of **3** at the TCB/HOPG interface (a: $c = 10^{-4}$ M, 100 × 100 nm^2, $V_s = -0.8$ V, $I_t = 34$ pA; b: $c = 10^{-3}$ M, 124 × 124 nm^2, $V_s = -0.8$ V, $I_t = 34$ pA, c: 33.9 × 33.9 nm^2; $c = 10^{-2}$ M; $V_S = -0.9$ V; $I_t = 52$ pA; all samples thermally annealed for 20 s at 80 °C; unit cell: $a = b = (2.0 \pm 0.1)$ nm, $\gamma(a,b) = (60 \pm 2)°$); (d) proposed supramolecular model. Red and white lines indicate unit cell and HOPG main axis directions, respectively.

2.5 Additional STM images of 1a/1b and 2

A nanopattern of **1a** was formed (by adding a solution of **1a** to HOPG at an elevated temperature of 80 °C), allowed to cool to rt, and an identical volume of a 10^{-6} M solution of **2** is added. The scanning tunneling microscopy images of this sample shows that **2** intercalates into **1a**.

Regarding the overview image shown in Figure S8c **2**, at an added concentration of 10^{-6} M*,* about 17% of all intramolecular pores are filled with Kekulene. As seen in Figure S6, **2** forms self-assembled monolayers of its own at certain concentrations. Increasing the concentration of **2** in the pore-filling experiment has been unsuccessful so far, as **2** tends to adsorb competitively in the mixture thus disrupting the stability of the template.

Analogous intercalation experiments have been carried out with **1b** as a template, resulting in a similar pore-filling phenomenon. A nanopattern of **1b** was formed (by adding a solution of **1b** to HOPG at an elevated temperature of 80 °C), allowed to cool to rt, and an identical volume of a 10^{-6} M solution of **2** is added. The scanning tunneling microscopy image in Figure S8g shows that **2** intercalates into **1b**. However, due to the longer alkoxy side chain periphery of **1a** on two opposing sides of the macrocycle in comparison to **1b**, the packing of the wide polymorph of **1a** tends to be more robust. As such, further intercalation experiments have been carried out with the more manageable template.

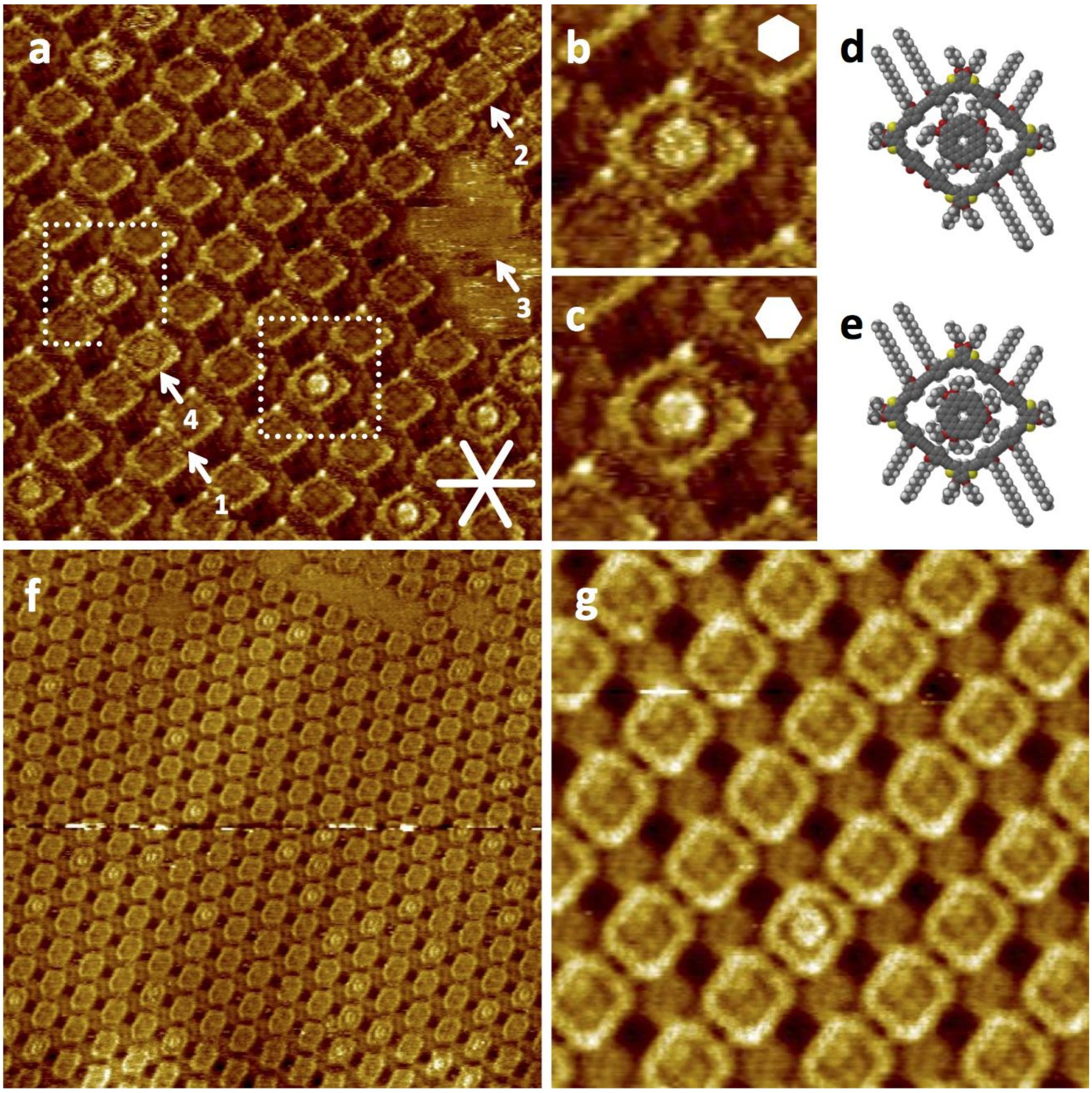

Figure S8: (a) Overview scanning tunneling microscopy image of a nanopattern of **1a** with intermolecularly intercalated **2** at the TCB/HOPG interface, (b)–(c) enlarged cutouts of the marked regions of (a), and (d)–(e) molecular models of the structures observed in (b) and (c), (f) overview scanning tunneling microscopy image of a nanopattern of **1a** with intermolecularly intercalated **2** at the TCB/HOPG interface, (g) overview scanning tunneling microscopy image of **1b** with intercalated **2** at the TCB/HOPG interface. Image parameters and sample preparation: (a) 1 µL *c*(**1a**) = 5×10^{-7} M (thermally annealed for 20 s at 80 °C), and 1 µL *c*(**2**) = 1×10^{-6} M added at rt; $V_S = -0.7$ V, $I_t = 49$ pA; 39.5 × 39.5 nm^2; (b) and (c): 8.7 × 8.7 nm^2; (f) 1 µL *c*(**1a**) = 5×10^{-7} M (thermally annealed for 20 s at 80 °C), and 1 µL *c*(**2**) = 1×10^{-6} M added at rt; $V_S = -0.8$ V, $I_t = 49$ pA; 98.6 × 98.6 nm^2; (g) 1 µL *c*(**1b**) = 5×10^{-7} M (thermally annealed for 20 s at 80 °C), and 1 µL *c*(**2**) = 1×10^{-6} M added at rt; $V_S = -0.7$ V, $I_t = 39$ pA; 25.0 × 25.0 nm^2. White lines indicate the HOPG main axis directions.

2.6 Additional STM images of 1a and 3

A nanopattern of **1a** was formed (by adding a solution of **1a** to HOPG at an elevated temperature of 80 °C), allowed to cool to rt, and an identical volume of a 10^{-4} M solution of **3** is added. The scanning tunneling microscopy images of this sample shows that **3** intercalates into **1a**.

Figure S9a shows two different polymorphs of **1a** indicated by dotted white lines. Bright dots in the center of a molecular square appear due to intercalated **3**. Filled and unfilled nanopores are clearly distinguishable. Polymorph A is favored for the intercalation of **3** into the intramolecular nanopores. Regarding the overview images shown in Figure S9a-b, roughly 3 % of the intramolecular nanopores of the wide polymorph are filled with octulene. For the more dense polymorph only few intercalations are observed. Again, we assume that this preference occurs due to interdigitating side chains inside the macrocycles.

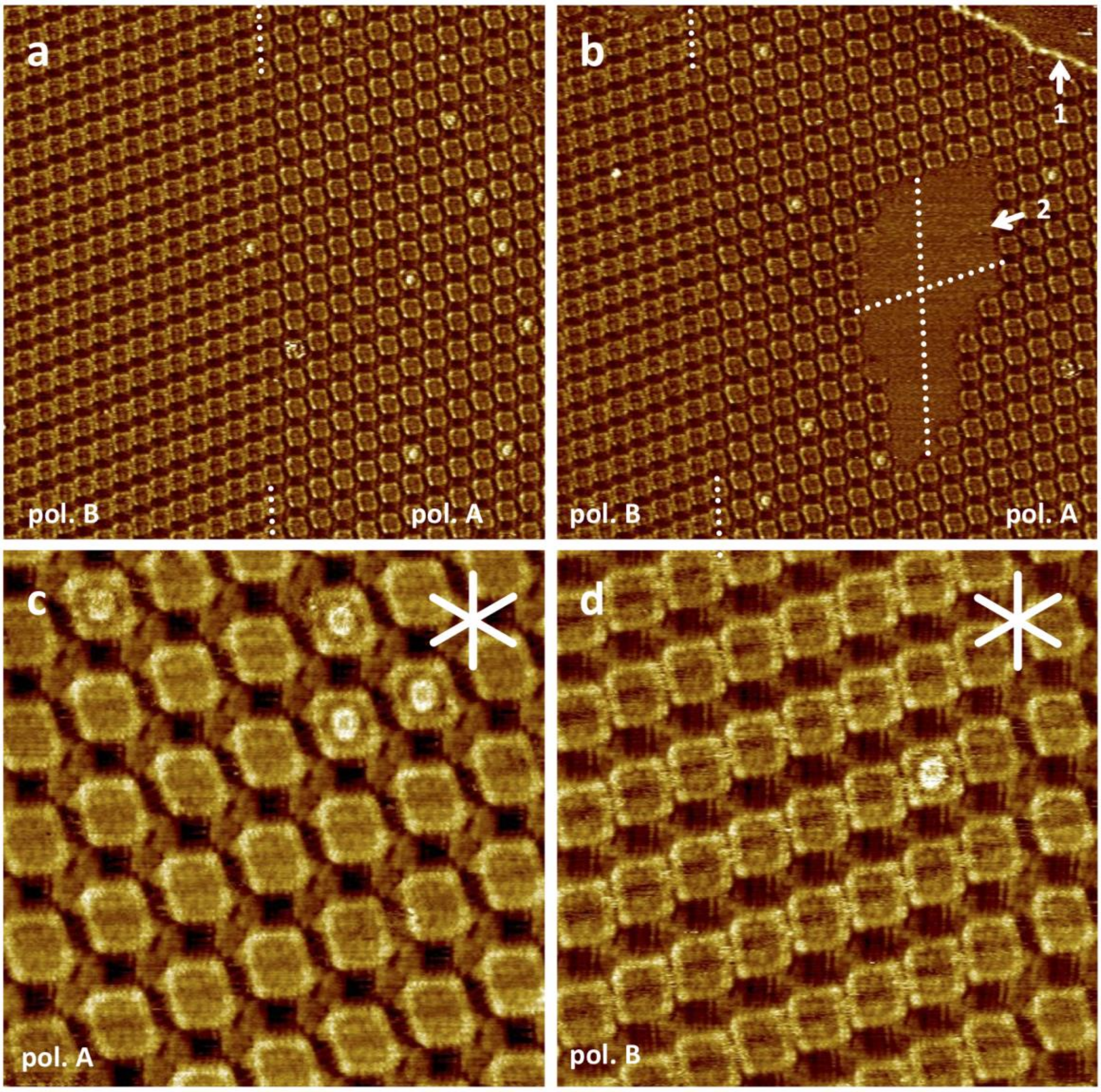

Figure S9: Scanning tunneling microscopy images of nanopatterns of **1a** on HOPG $c(\mathbf{1a}) = 5 \times 10^{-7}$ M (thermally annealed to 80 °C for 20 s), to which a solution of **3** $c(\mathbf{3}) = 1 \times 10^{-4}$ M at rt was applied. Image parameters: a: 147 × 147 nm^2, $V_s = -0.8$ V, $I_t = 34$ pA; b: 147 × 147 nm^2, $V_s = -0.8$ V, $I_t = 34$ pA; c: 31.2 × 31.2 nm^2, $V_s = -0.5$ V, $I_t = 24$ pA; d: 34.6 × 34.6 nm^2, $V_s = -0.8$ V, $I_t = 34$ pA.

Judging by the overview image shown in Figure S10b about 30% of all template molecules are occupied by octulene when increasing the concentration of **3** by a factor of 10. The high-resolution STM-image shown in Figure S10a also supports the intercalation model proposed in the main text. Further increase of the concentration of the added solution of **3** (to 10^{-2} M) again results in a higher degree of nanopore occupation by **3** (Figure S10c, 66% of local nanopores occupied).

Comparing the degree of occupation of **2** at a concentration of *c*(**2**) = 1×10^{-6} M (Figure S8) and **3** at a concentration of *c*(**3**) = 1×10^{-4} M (Figure S9) into a template of **1a** shows that even at a higher concentration of **3**, less pores are occupied. However, **3** makes it feasible to increase the concentration further without disrupting the template itself. This results in a higher degree of occupation into the template than what has been achievable with **2**.

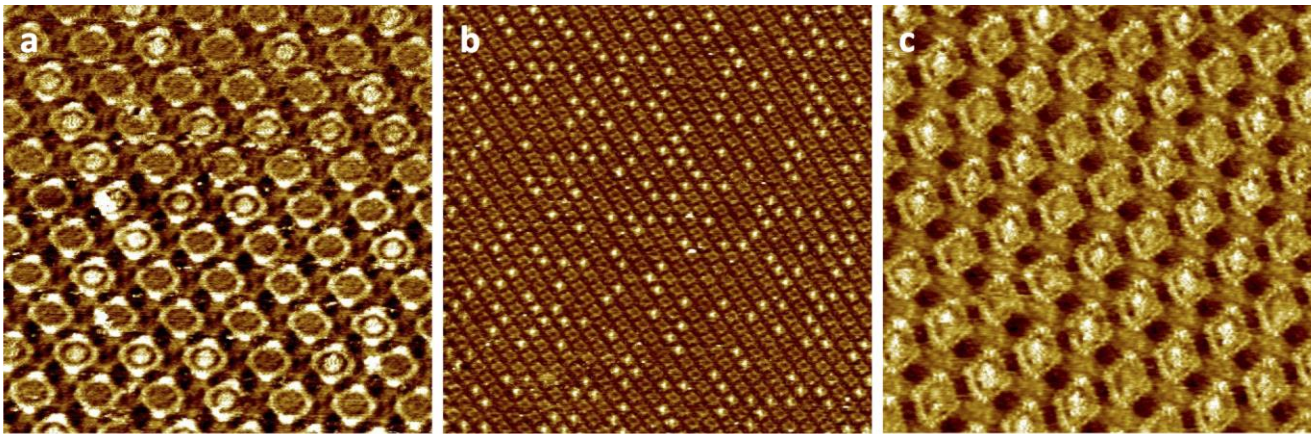

Figure S10: Scanning tunneling microscopy images of nanopatterns of **1a** on HOPG, *c*(**1a**) = 5×10^{-7} M (thermally annealed to 80 °C for 20 s), to which a solution of **3** (a/b) with *c*(**3**) = 1×10^{-3} M or (c) with *c*(**3**) = 1×10^{-2} M at rt was applied. Image parameters: a: 42.5 × 42.5 nm^2, V_s = −1.0 V, I_t = 44 pA; b: 158 × 158 nm^2, V_s = −0.7 V, I_t = 44 pA; c: 37.6 × 37.6 nm2, V_s = −0.4 V, I_t = 62 pA.

3. Synthesis

3.1 Synthetical strategy

Here we present the syntheses of arylene–alkynylene macrocycles, in which four linear phenylene–ethynylene–butadiynylene (PEB) rigid rods are connected by four dithiophene corner building blocks. The molecules have quadratic backbones, and each PEB unit is substituted with two alkoxy side chains. A pair of (long) $OC_{16}H_{33}$ side chains is attached to two opposite PEB units, and another pair of (shorter) OC_nH_{2n+1} (n = 6, 10) side chains is attached to the two other PEB units. Therefore, the backbone has a D_{4h} symmetry, whereas the symmetry of the whole molecule is reduced to D_{2h} as a consequence of the substitution pattern. Moreover, each of the four dithiophene corner building blocks is substituted with two OC_4H_9 side chains, which improve the compound solubility in common organic solvents.

The diiodo-substituted corner building block **5** (Scheme S1) was obtained from the dithiophene building block **4**, which has been previously described [1]. The synthesis of the $OC_{10}H_{25}$ and OC_6H_{13}-substituted rigid rod segments **9a/b** started from **6**, which has been described previously [2]. Compound **6** was coupled through a Williamson ether synthesis or under Mitsunobu coupling conditions with the appropriate alkane derivatives to yield **7a/b**. These were consecutively functionalized with (1st) trimethylsilyl acetylene (TMS-acetylene) and (2nd) [(3-cyanopropyl)dimethylsilyl]acetylene (CPDIPS-acetylene) under palladium catalyzed Sonogashira coupling conditions to yield **8a/b** [3,4]. Both reactions were performed in a one-pot reaction, and the intermediates were not isolated/purified. Deprotection of the TMS protective group of **8a/b** using K_2CO_3 in MeOH yielded the mono-CPDIPS-protected bisacetylenes **9a/b**.

The synthesis of the bis-CPDIPS-protected bis-$OC_{16}H_{33}$-substituted PEB **10** has been previously described [5]. Deprotection of the CPDIPS units using tetrabutylammonium fluoride (TBAF)/H_2O yielded the bisacetylene **11**, which was coupled with two diiodinated corner building blocks **5** under Palladium catalyzed Sonogashira coupling conditions to yield the diiodo-substituted corner-side-corner segment **12**. Sonogashira coupling conditions were used (again) to react **12** with the alkoxy-substituted PEB segments **9a/b** to yield the bis-CPDIPS-protected half-ring structures **13a/b**. After a deprotection of both CPDIPS groups (that was performed by using TBAF/H_2O again), the bisacetylenes **14a/b** were obtained. These were cyclooligomerized under palladium-catalyzed oxidative coupling conditions (using a high-dilution strategy) to yield arylene–alkynylene macrocycles. From the crude product mixture, **1a/b** were isolated using recycling gel permeation chromatography (recGPC).

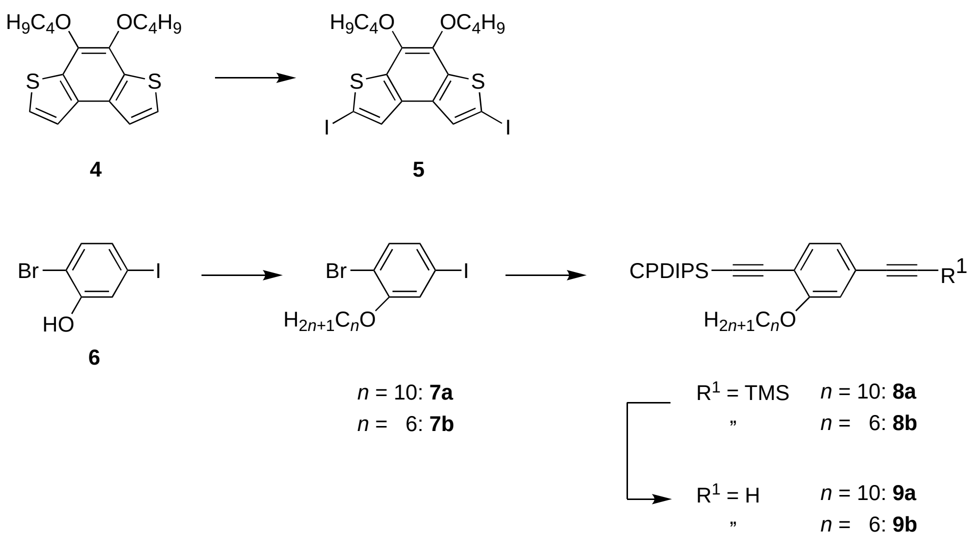

Scheme S1: Overview synthesis (I).

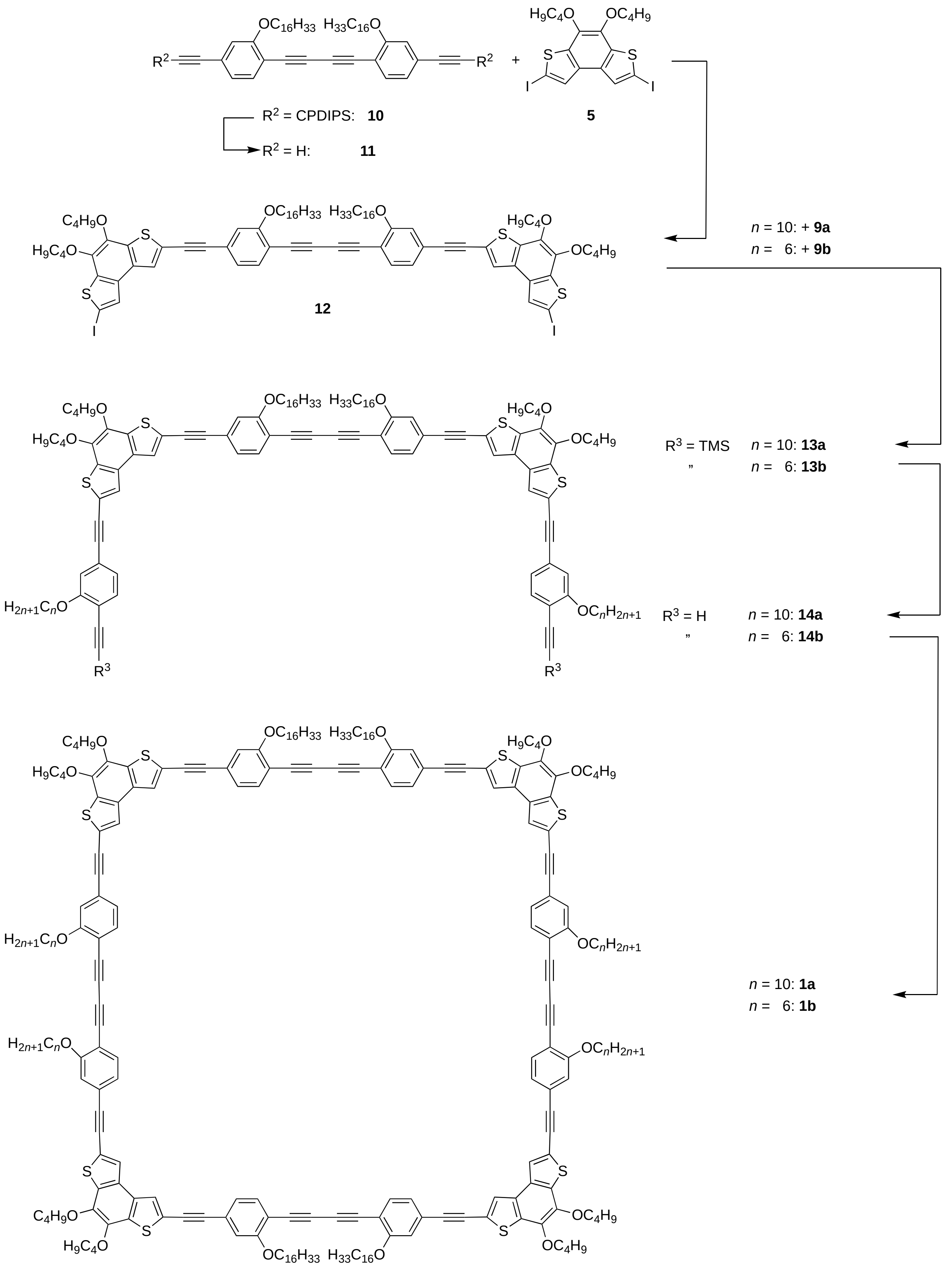

Scheme S2: Overview synthesis (II).

3.2 Synthesis of the condensed dithiophene corner building block 5

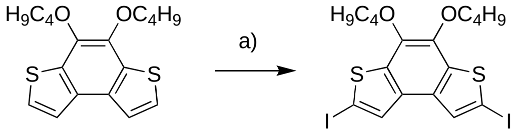

Scheme S3: Synthesis of the corner building block **5**. Conditions: a) *n*-BuLi, TMEDA, I_2, −78 °C to rt, 51%.

4 was synthesized as previously described [1].

5

Under an argon atmosphere, **4** (675 mg, 2.02 mmol) was dissolved in dry THF (20 mL) and cooled to −78 °C. TMEDA (1.22 mL, 8.08 mmol) and *n*-BuLi (1.6 M in hexane, 5.05 mL, 8.08 mmol) were added, and the solution was stirred for 30 min at −78 °C, before a solution of I_2 (1.28 g, 10.1 mmol) in dry THF (3 mL) was added. The reaction mixture was allowed to warm to rt overnight and subsequently was diluted with water and DCM. The organic layer was separated, and the aqueous phase was extracted with DCM two times. The combined organic layers were washed with water, aqueous Na_2SO_3 solution (10%), aqueous HCl (1 mol/L), and brine. The organic phase was dried over Na_2SO_4, and the solvent was evaporated. The crude product was purified by column chromatography using CH as eluent (R_f = 0.37) to yield **5** as crystalline, brightly yellow solid (602 mg, 51%). Chemical formula: $C_{18}H_{20}I_2O_2S_2$. Molecular weight: 586.29 g/mol. ^{1}H NMR (300.1 MHz, $CDCl_3$, 298 K) δ [ppm] = 7.71 (s, 2H), 4.21 (t, J = 6.5 Hz, 4H), 1.83-1.74 (m, 4H), 1.61-1.49 (m, 4H), 1.00 (t, J = 7.4 Hz, 6H). ^{13}C NMR (75.5 MHz, $CDCl_3$, 298 K) δ [ppm] = 141.2, 138.2, 131.8, 130.9, 73.5, 32.4, 19.2, 13.9. MS (EI, 70eV) *m/z* (%) = 586.0 (69.1) $[M]^{+\cdot}$, 472.8 (100); EI HRMS: *m/z* $[M]^{+\cdot}$ (calcd: 585.8989 g/mol) found: 585.8990.

3.3 Synthesis of the rigid rod segments 9a/b

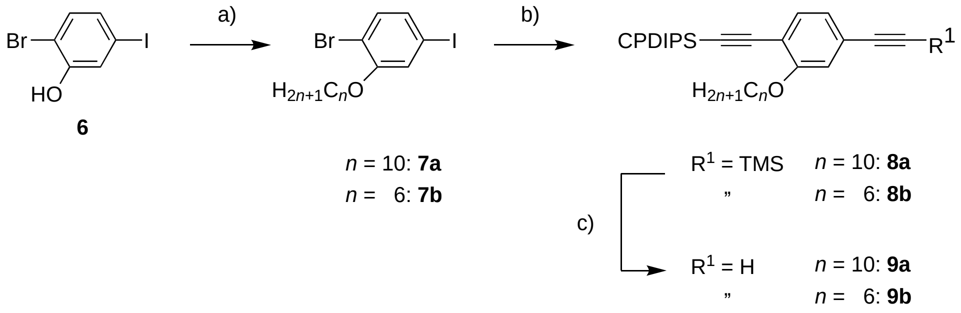

Scheme S4: Synthesis of **9a/b**. Conditions: a) *n* = 10:1-decanol, PPh_3, diisopropylazodicarboxylate (DIAD), THF, 0 °C–rt, 1 d, 78%; *n* = 6:1-bromhexane, K_2CO_3, KI, DMF, 60 °C, 1 d, 88%; b) trimethylsilyl acetylene (TMS-acetylene), $Pd(PPh_3)_2Cl_2$, PPh_3, CuI, piperidine, THF, rt, 6 h, 55 °C, [(3-cyanopropyl)dimethylsilyl]acetylene (CPDIPS-acetylene), 16 h; **8a**: 74%; **8b**: 99%; c) K_2CO_3, THF, MeOH, rt, 3 h; **9a**: 81%; **9b**: 63%.

2-Bromo-5-iodophenol (**6**) was obtained as described previously [1].

7a

Under an argon atmosphere, **6** (114 mg, 381 µmol), triphenylphosphane (PPh_3, 120 mg, 458 µmol) and 1-decanol (60.3 mg, 381 µmol) were suspended in dry THF (20 mL) and cooled to 0 °C. Diisopropylazodicarboxylate (DIAD, 108 mg, 0.1 mL, 533 µmol) in dry THF (1 mL) was added, and the reaction mixture was stirred overnight while allowing it to warm to rt. The reaction mixture was then diluted with water and diethyl ether. The organic layer was separated, and the aqueous layer was extracted with diethyl ether. The combined organic layers were washed with brine and dried over Na_2SO_4, before the solvent was evaporated. Purification by column chromatography on silica gel using CH (R_f = 0.63) yielded **7a** as white solid (130 mg, 296 µmol, 78%). Chemical formula: $C_{16}H_{24}BrIO$. Molecular weight: 439.18 g/mol. ^{1}H NMR (300 MHz, C*D*Cl$_3$, RT): δ [ppm] = 7.23 (d, 3J = 8.1 Hz, 1H), 7.15 (d, 4J = 1.8 Hz, 1H), 7.18 – 7.08 (m, 1H), 3.98 (t, 3J = 6.5 Hz, 2H), 1.89 – 1.77 (m, 2H), 1.53 – 1.42 (m, 2H), 1.43 – 1.22 (m, 12H), 0.93 – 0.84 (m, 3H). ^{13}C NMR (76 MHz, C*D*Cl$_3$, RT): δ [ppm] = 156.27, 134.59, 130.77, 122.43, 112.48, 92.45, 69.63, 32.06, 29.69, 29.47, 29.42, 29.10, 26.06, 22.84, 14.28. MS (EI, 70 eV): *m/z* (%): 438.0 (34) [M]+· ; 297.9 (100) $[M-C_{10}H_{20}]^+$.

7b

Under an argon atmosphere, **6** (312 mg, 1.04 mmol), 1-bromohexane (215 mg, 1.30 mmol), K_2CO_3 (575 mg, 4.16 mmol), and KI (17.2 mg, 104 µmol) were suspended in dry DMF (15 mL) and stirred at 60 °C for 25 h. After cooling to rt, the reaction mixture was diluted with DCM and water. The organic layer was separated and the aqueous layer was extracted with DCM. The combined organic layers were washed with aqueous HCl (10%), water, and brine, and dried over Na_2SO_4, before the solvent was evaporated. Purification by column chromatography on silica gel using CH (R_f = 0.66) yielded **7b** as colorless viscous liquid (350 mg, 914 µmol, 88%). Chemical formula: $C_{12}H_{16}BrIO$. Molecular weight: 383.07 g/mol. ^{1}H NMR (400 MHz, C*D*Cl$_3$, RT): δ [ppm] = 7.27 (d, ^3J = 8.2 Hz, 1H), 7.20 (d, ^4J = 1.9 Hz, 1H), 7.17 (dd, ^3J = 8.2 Hz, ^4J = 1.9 Hz, 1H), 4.03 (t, ^3J = 6.5 Hz, 2H), 1.92 – 1.82

(m, 2H), 1.60 – 1.50 (m, 2H), 1.44 – 1.36 (m, 4H), 0.99 – 0.93 (m, 3H). ^{13}C NMR (101 MHz, C*D*Cl$_3$, RT): δ [ppm] = 156.26, 134.59, 130.77, 122.43, 112.48, 92.45, 69.62, 31.59, 29.07, 25.74, 22.71, 14.16. MS (EI, 70 eV): *m/z* (%): 381.9 (30) $[M]^{+\cdot}$; 297.8 (100) $[M\text{-}C_6H_{12}]^+$.

8a

Under an argon atmosphere, trimethylsilylacetylene (TMS-acetylene, 29.8 mg, 304 µmol) was added to a solution of **7a** (127 mg, 289 µmol), $Pd(PPh_3)_2Cl_2$ (6.09 mg, 8.67 µmol), PPh_3 (6.06 mg, 23.1 µmol), CuI (2.75 mg, 14.5 µmol) in piperidine (10 mL). The solution was stirred for 16 h at rt. Subsequently, the solution was heated to 55 °C, [(3-cyanopropyl)dimethylsilyl]acetylene (CPDIPS-acetylene, 180 mg, 867 µmol)[4] was added, and the reaction mixture was stirred at 55 °C for another 24 h. The suspension was diluted with diethyl ether and water. The organic layer was separated, and the aqueous layer was extracted with diethyl ether. The combined organic layers were washed with water, aqueous HCl (10%), and brine, dried with Na_2SO_4, and the solvent was evaporated. Purification by column chromatography on silica gel using CH/DCM 2:1 as eluent (R_f = 0.25) yielded **8a** (114 mg, 214 µmol, 74%) as colorless viscous liquid. Chemical formula: $C_{33}H_{53}NOSi_2$. Molecular weight: 535.96 g/mol. ^{1}H NMR (400 MHz, C*D*Cl$_3$, RT): δ [ppm] = 7.32 (d, ^3J = 7.8 Hz, 1H), 6.98 (dd, ^3J = 7.8 Hz, ^4J = 1.4 Hz, 1H), 6.91 (d, ^4J = 1.4 Hz, 1H), 3.98 (t, ^3J = 6.4 Hz, 2H), 2.42 (t, ^3J = 7.0 Hz, 2H), 1.93 – 1.74 (m, 4H), 1.52 – 1.43 (m, 2H), 1.38 – 1.22 (m, 12H), 1.15 – 1.03 (m, 14H), 0.91 – 0.86 (m, 3H), 0.85 – 0.79 (m, 2H), 0.25 (s, 9H). ^{13}C NMR (101 MHz, C*D*Cl$_3$, RT): δ [ppm] = 159.99, 133.62, 124.51, 124.05, 114.85, 113.11, 104.88, 103.86, 96.03, 95.36, 68.70, 32.04, 29.80, 29.76, 29.58, 29.50, 29.41, 26.24, 22.83, 21.46, 20.90, 18.37, 18.13, 14.27, 11.95, 9.76, 0.07. MS (EI, 70 eV): *m/z* (%): 535.3 (7) $[M]^{+\cdot}$; 492.3 (100) $[M\text{-}C_3H_7]^+$.

8b

Under an argon atmosphere, trimethylsilylacetylene (TMS-acetylene, 85.6 mg, 872 µmol) was added to a solution of **7b** (318 mg, 830 µmol), $Pd(PPh_3)_2Cl_2$ (17.5 mg, 24.9 µmol), PPh_3 (17.4 mg, 66.4 µmol), CuI (7.90 mg, 41.5 µmol) in piperidine (13 mL). The solution was stirred for 16 h at rt. Subsequently, the solution was heated to 55 °C, [(3-cyanopropyl)dimethylsilyl]acetylene (CPDIPS-acetylene, 516 mg, 2.49 mmol) was added, and the reaction mixture was stirred for another 24 h. The suspension was diluted with diethyl ether and water. The organic layer was separated, and the aqueous layer was extracted with diethyl ether. The combined organic layers were washed with water, aqueous HCl (10%), and brine, dried with Na_2SO_4, and the solvent was evaporated. Purification by column chromatography on silica gel using CH/DCM 2:1 as eluent (R_f = 0.24) yielded **8b** (396 mg, 825 µmol, 99%) as colorless viscous liquid. Chemical formula: $C_{29}H_{45}NOSi_2$. Molecular weight: 479.86 g/mol. ^{1}H NMR (500 MHz, C*D*Cl$_3$, RT): δ [ppm] = 7.32 (d, ^3J = 7.8 Hz, 1H), 6.97 (dd, ^3J = 7.8 Hz, ^4J = 1.4 Hz, 1H), 6.91 (d, ^4J = 1.3 Hz, 1H), 3.98 (t, ^3J = 6.4 Hz, 2H), 2.42 (t, ^3J = 7.0 Hz, 2H), 1.91 – 1.75 (m, 4H), 1.52 – 1.44 (m, 2H), 1.38 – 1.28 (m, 4H), 1.14 – 1.03 (m, 14H), 0.93 – 0.88 (m, 3H), 0.85 – 0.79 (m, 2H), 0.25 (s, 9H). ^{13}C NMR (126 MHz, C*D*Cl$_3$, RT): δ [ppm] = 159.97, 133.63, 124.51, 124.04, 119.92, 114.84, 113.10, 104.87, 103.85, 96.03, 95.36, 68.69, 31.73, 29.36, 27.06, 25.91, 22.80, 21.45, 20.89, 18.36, 18.18, 18.12, 17.95, 14.22, 11.94, 9.76, 0.07. MS (EI, 70 eV): *m/z* (%): 290.0 (100).

9a

Under an argon atmosphere, K_2CO_3 (88.4 mg, 640 µmol) and methanol (10 mL) were added to a solution of **8a** (114 mg, 213 µmol) in dry THF (10 mL). The reaction mixture was stirred for 3 h at rt, before water and diethyl ether were added. The organic layer was separated, and the aqueous layer was extracted with diethyl ether. The combined organic layers were washed with water and brine, and dried over Na_2SO_4, before the solvent was evaporated. Column chromatography using silica gel and CH/DCM 2:1 as eluent (R_f = 0.24) yielded **9a** (114 mg, 213 µmol, 81%) as colorless viscous liquid. Chemical formula: $C_{30}H_{45}NOSi$. Molecular weight: 463.78 g/mol. ^{1}H NMR (400 MHz, C*D*Cl$_3$, RT): δ [ppm] = 7.35 (d, ^3J = 7.8 Hz, 1H), 7.00 (dd, ^3J = 7.8 Hz, ^4J = 1.4 Hz, 1H), 6.94 (d, ^4J = 1.4 Hz, 1H), 3.98 (t, ^3J = 6.4 Hz, 2H), 3.14 (s, 1H), 2.42 (t, ^3J = 7.0 Hz, 2H), 1.93 – 1.75 (m, 4H), 1.53 – 1.43 (m, 2H), 1.38 – 1.21 (m, 12H), 1.15 – 1.05 (m, 14H), 0.91 – 0.86 (m, 3H), 0.85 – 0.79 (m, 2H). ^{13}C NMR (101 MHz, C*D*Cl$_3$, RT): δ[ppm] = 160.03, 133.72, 124.13, 123.44, 119.90, 115.07, 113.53, 103.65, 95.54, 83.56, 78.70, 68.73, 32.04, 29.79, 29.76, 29.57, 29.49, 29.39, 26.23, 22.83, 21.46, 20.88, 18.36, 18.12, 14.27, 11.94, 9.74. MS (EI, 70 eV): *m/z* (%): 463.3 (4) $[M]^{+\cdot}$; 420.3 (100) $[M-C_3H_7]^+$.

9b

Under an argon atmosphere, K_2CO_3 (342 mg, 2.48 mmol) and methanol (15 mL) were added to a solution of **8b** (396 mg, 825 µmol) in dry THF (15 mL). The reaction mixture was stirred for 3 h at rt, before water and diethyl ether were added. The organic layer was separated, and the aqueous layer was extracted with diethyl ether. The combined organic layers were washed with water and brine, and dried over Na_2SO_4, before the solvent was evaporated. Column chromatography using silica gel and CH/DCM 2:1 as eluent (R_f = 0.24) yielded **9b** (211 mg, 520 µmol, 63%) as colorless viscous liquid. Chemical formula: $C_{26}H_{37}NOSi$. Molecular weight: 407.67 g/mol. ^{1}H NMR (400 MHz, C*D*Cl$_3$, RT): δ [ppm] = 7.35 (d, ^3J = 7.8 Hz, 1H), 7.01 (dd, ^3J = 7.9 Hz, ^4J = 1.4 Hz, 1H), 6.94 (d, ^4J = 1.3 Hz, 1H), 3.98 (t, ^3J = 6.4 Hz, 2H), 3.15 (s, 1H), 2.42 (t, ^3J = 6.9 Hz, 2H), 1.93 – 1.76 (m, 4H), 1.53 – 1.44 (m, 2H), 1.37 – 1.28 (m, 4H), 1.16 – 1.02 (m, 14H), 0.94 – 0.88 (m, 3H), 0.86 – 0.79 (m, 2H). ^{13}C NMR (101 MHz, C*D*Cl$_3$, RT): δ[ppm] = 160.02, 133.73, 124.13, 123.44, 119.90, 115.07, 113.53, 103.65, 95.53, 83.55, 78.71, 68.72, 31.73, 29.34, 25.90, 22.79, 21.45, 20.88, 18.35, 18.11, 14.21, 11.93, 9.73. MS (EI, 70 eV): *m/z* (%): 407.3 (4) $[M]^{+\cdot}$; 364.2 (100) $[M-C_3H_7]^+$.

3.4 Synthesis of the macrocycles 1a/b

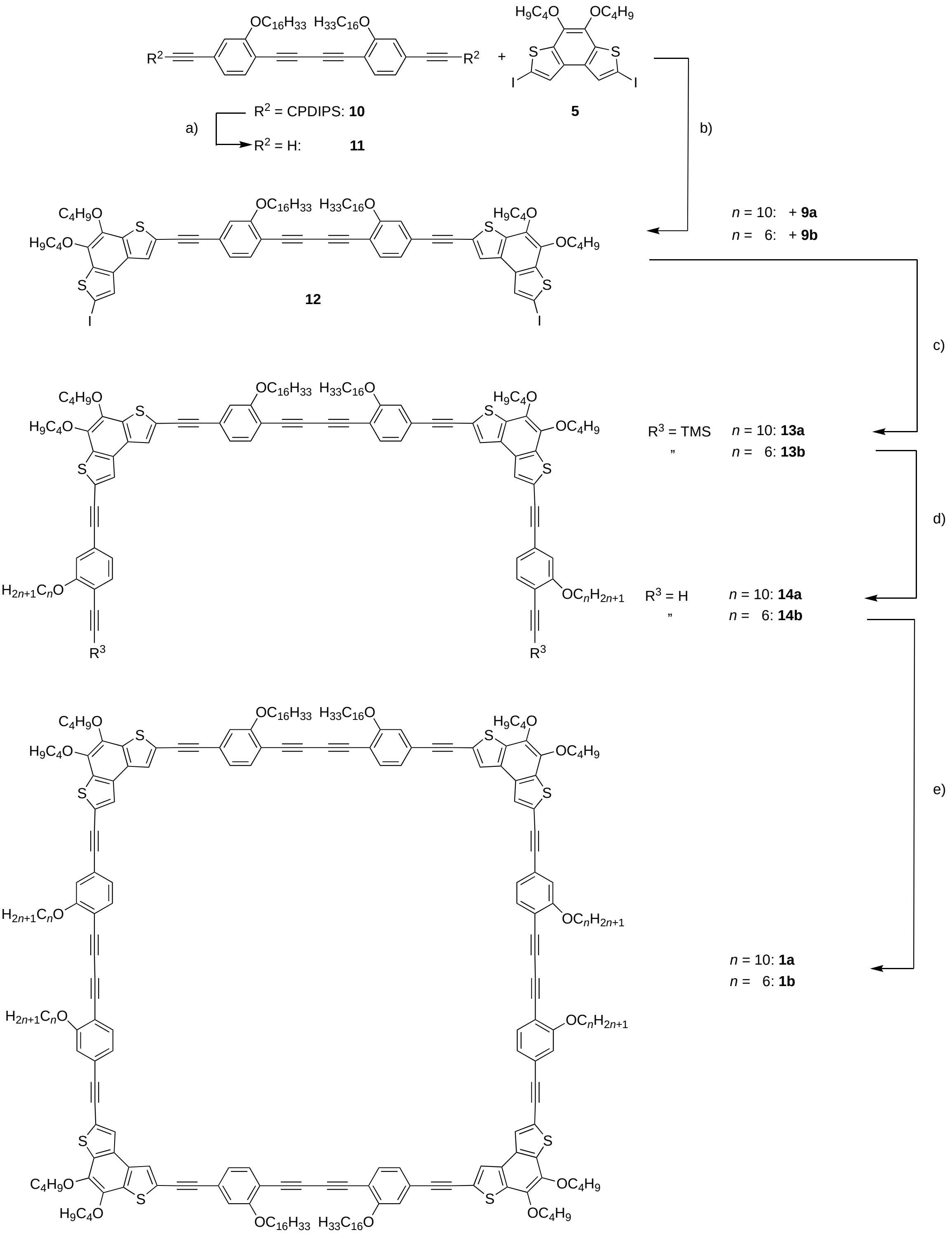

Scheme S5: Synthesis of **1a/b**: Conditions: a) TBAF, H_2O, THF, rt, 3 h, 27%; b) $Pd(PPh_3)_2Cl_2$, PPh_3, CuI, piperidine, THF, 40 °C, 48 h, 27%; c) *n* = 10, 6: $Pd_2(dba)_3$, PPh_3, CuI, piperidine, THF, 60 °C, 6 h, **13a**: 69%; **13b**: 69%; d) TBAF, THF, rt, 3 h, **14a**: 90%; **14b**: 84%; e) $Pd(PPh_3)_2Cl_2$, CuI, I_2, THF, iPr_2NH, THF, 50 °C, 72 h; **1a:** 27%; **1b**: 13%.

10 was synthesized as previously described [5].

11

10 (193 mg, 0.176 mmol) was dissolved in THF (15 mL), the solution was cooled to 0 °C, and tetrabutylammonium fluoride (TBAF, 0.18 mL, 0.18 mmol, 1 M solution in THF) and water (0.88 mL) were added. The progress of deprotection was controlled via thin layer chromatography (TLC), and after 3 h the reaction was diluted with water. After phase separation, the aqueous layer was extracted with DCM. The combined organic layers were washed with water, aqueous HCl (0.5 M), and brine. After drying over Na_2SO_4 and evaporating the solvent under reduced pressure, the crude product was purified by column chromatography using silica gel and CH/DCM 4:1 as eluent (R_f = 0.58) to yield **11** as yellow solid (27%, 34 mg, 47 µmol). Chemical formula: $C_{52}H_{74}O_2$. Molecular weight: 731.14 g/mol. 1H NMR (400.1 MHz, CDCl3, 298 K) δ [ppm] = 7.40 (d, J = 8.0 Hz, 2H), 7.02 (dd, J = 7.8, 1.4 Hz, 2H), 6.97 (d, J = 1.2 Hz, 2H), 4.02 (t, J = 6.6 Hz, 4H), 3.18 (s, 2H), 1.88-1.81 (m, 4H), 1.53-1.45 (m, 4H), 1.40-1.24 (m, 48H), 0.88 (t, J = 6.8 Hz, 6H). ^{13}C NMR (100.6 MHz, CDCl3, 298 K) δ [ppm] = 160.5, 134.1, 124.2, 123.8, 115.3, 112.6, 83.3, 79.5, 79.1, 78.8, 69.0, 31.9, 29.7, 29.7, 29.6, 29.6, 29.4, 29.3, 28.9, 25.9, 22.7, 14.1. MS (EI, 70eV) *m/z* (%) = 730.5 (40) $[M]^{+\cdot}$, 519.3 (100) $[M-C_{15}H_{31}]^+$; EI HRMS: *m/z* $[M]^{+\cdot}$ (calcd: 730.5683 g/mol) found: 730.5684.

12

Under an argon atmosphere, **5** (497 mg, 848 µmol), $Pd(PPh_3)_2Cl_2$ (0.89 mg, 1.3 µmol), PPh_3 (0.89 mg, 3.4 µmol) and CuI (0.40 mg, 2.1 µmol) were dissolved in dry THF (50 mL) and dry piperidine (30 mL) and heated to 40 °C. A solution of **11** (62 mg, 85 µmol) in dry THF (40 mL) was added to the reaction mixture over 48 h. The reaction mixture was diluted with DCM and water, and the organic layer was extracted with DCM. The combined organic layers were washed with water, aqueous acetic acid (10%, 3×), aqueous NaOH (1 M, 2×), and brine (2×), and dried over Na_2SO_4, before the solvent was evaporated under reduced pressure. The crude product was purified by (short filtration) column chromatography using silica gel and CH/DCM 1:2 as eluent (R_f = 0.35), and by recycling gel permeation chromatography (recGPC) to yield **12** (38 mg, 23 µmol, 27%) as yellow solid. Chemical formula: $C_{88}H_{112}I_2O_6S_4$. Molecular weight: 1647.90 g/mol. ^{1}H NMR (400.1 MHz, C*D*Cl$_3$, 298 K) δ [ppm] = 7.75 (s, 2H), 7.71 (s, 2H), 7.46 (d, J = 8.0 Hz, 2H), 7.09 (dd, J = 8.0, 1.2 Hz, 2H), 7.04 (d, J = 1.2 Hz, 2H), 4.27-4.22 (m, 8H), 4.08 (t, J = 6.6 Hz, 4H), 1.92-1.77 (m, 12H), 1.63-1.49 (m, 12H), 1.43-1.24 (m, 48H), 1.01 (t, J = 7.4 Hz, 6H), 1.01 (t, J = 7.4 Hz, 6H), 0.86 (t, J = 6.8 Hz, 6H). ^{13}C NMR (75.5 MHz, C*D*Cl$_3$, 298 K) δ [ppm] = 160.7, 142.2, 141.7, 138.6, 134.6, 134.2, 132.2, 132.0, 129.1, 126.9, 124.3, 123.6, 122.4, 114.5, 112.4, 94.8, 85.2, 79.8, 79.3, 76.9, 73.6, 73.5, 69.1, 32.4, 31.9, 29.7, 29.7, 29.7, 29.6, 29.4, 29.0, 25.9, 22.7, 19.3, 19.3, 14.1, 13.9. MS (MALDI-TOF) *m/z* (%) = 1896.6 (19) $[M+Ma]^+$, 1646.6 (100) $[M]^+$, 1520.8 (10) $[C_{88}H_{113}IO_6S_4]^+$.

13a

Under an argon atmosphere, **12** (80 mg, 48.5 µmol), $Pd_2(dba)_3$, 1.33 mg, 1.46 µmol), PPh_3 (0.51 mg, 1.94 µmol), and CuI (0.46 mg, 2.43 µmol) were dissolved in dry piperidine (10 mL) and heated to 55 °C. **9a** (71.0 mg, 153 µmol) in THF (5 mL) was added, and the reaction mixture was stirred for 19 h at this temperature. The reaction mixture was diluted with water and diethyl ether, the aqueous layer was extracted with diethyl ether, and the combined organic phases were washed with water, aqueous HCl (10%), water, and brine, and dried over

Na_2SO_4 before the solvent was evaporated under reduced pressure. Purification by column chromatography on silica gel using CH/DCM 3:2 (R_f = 0.33) as eluent yielded **13a** as yellow solid (78.5 mg, 33.8 µmol, 69%). Chemical formula: $C_{148}H_{200}N_2O_8S_4Si_2$. Molecular weight: 2319.64 g/mol. ^{1}H NMR (400 MHz, C*D*Cl$_3$, RT): δ [ppm] = 7.76 (s, 2H), 7.75 (s, 2H), 7.47 (d, ^3J = 7.8 Hz, 2H), 7.41 (d, ^3J = 7.8 Hz, 2H), 7.12 – 7.07 (m, 4H), 7.05 (d, ^4J = 1.4 Hz, 2H), 7.02 (d, ^4J = 1.4 Hz, 2H), 4.27 (t, ^3J = 6.5 Hz, 8H), 4.08 (t, ^3J = 6.6 Hz, 4H), 4.04 (t, ^3J = 6.4 Hz, 4H), 2.44 (t, ^3J = 7.0 Hz, 4H), 1.95 – 1.79 (m, 20H), 1.66 – 1.48 (m, 16H), 1.39 – 1.20 (m, 72H), 1.17 – 1.06 (m, 28H), 1.05 – 0.98 (m, 12H), 0.93 – 0.81 (m, 16H). ^{13}C NMR (101 MHz, C*D*Cl$_3$, RT): δ [ppm] = 160.84, 160.16, 142.92, 142.88, 135.08, 135.03, 134.35, 133.86, 130.67, 130.66, 127.23, 127.11, 124.46, 123.96, 123.82, 123.58, 122.92, 122.82, 119.91, 114.67, 114.32, 113.37, 112.63, 103.84, 95.81, 95.17, 95.11, 85.38, 84.78, 80.00, 79.42, 73.72, 69.24, 68.80, 32.62, 32.10, 32.06, 29.89, 29.84, 29.82, 29.80, 29.78, 29.61, 29.54, 29.53, 29.51, 29.43, 29.14, 27.08, 27.07, 26.27, 26.11, 22.86, 22.85, 21.49, 20.91, 19.46, 18.39, 18.14, 14.28, 14.08, 11.97, 9.77. MS (MALDI-pos, DCTB): *m/z* (%): 2567.4 (35) [M+DCTB]$^+$; 2317.3 (100) [M]$^+$.

13b

Under an argon atmosphere, **12** (80 mg, 48.5 µmol), $Pd_2(dba)_3$, 1.33 mg, 1.46 µmol), PPh_3 (0.51 mg, 1.94 µmol), and CuI (0.46 mg, 2.43 µmol) were dissolved in dry piperidine (10 mL) and heated to 55 °C. **9b** (59.4 mg, 146 µmol) in dry THF (5 mL) was added dropwise, and the reaction mixture was stirred for 19 h at this temperature. The reaction mixture was diluted with water and diethyl ether, the aqueous layer was extracted with diethyl ether, and the combined organic phases were washed with water, aqueous HCl (10%), water, and brine, and dried over Na_2SO_4 before the solvent was evaporated under reduced pressure. Purification by column chromatography on silica gel using CH/DCM 3:2 (R_f = 0.33) as eluent yielded **13b** as yellow solid (73.8 mg, 33.5 µmol, 69%). Chemical formula: $C_{140}H_{184}N_2O_8S_4Si_2$. Molecular weight: 2207.43 g/mol. ^{1}H NMR (400 MHz, C*D*Cl$_3$, RT): δ [ppm] = 7.76 (s, 2H), 7.75 (s, 2H), 7.47 (d, ^3J = 7.9 Hz, 2H), 7.41 (d, ^3J = 7.9 Hz, 2H), 7.13 – 7.07 (m, 4H), 7.05 (d, ^4J = 1.3 Hz, 2H), 7.02 (d, ^4J = 1.3 Hz, 2H), 4.27 (t, ^3J = 6.5 Hz, 8H), 4.11 – 4.02 (m, 8H), 2.44 (t, ^3J = 7.0 Hz, 4H), 1.95 – 1.78 (m, 20H), 1.66 – 1.48 (m, 16H), 1.40 – 1.20 (m, 56H), 1.17 – 1.06 (m, 28H), 1.02 (t, ^3J = 7.3 Hz, 12H), 0.96 – 0.90 (m, 6H), 0.89 – 0.81 (m, 10H). ^{13}C NMR (101 MHz, C*D*Cl$_3$, RT): δ [ppm] = 160.84, 160.15, 142.92, 142.88, 135.09, 135.03, 134.35, 133.88, 130.68, 130.66, 127.23, 127.12, 124.47, 123.97, 123.82, 123.58, 122.93, 122.82, 119.92, 114.67, 114.32, 113.37, 112.64, 103.84, 95.81, 95.17, 95.11, 85.38, 84.79, 80.00, 79.42, 73.72, 69.25, 68.80, 32.63, 32.10, 31.77, 29.89, 29.85, 29.82, 29.80, 29.54, 29.53, 29.39, 29.15, 27.08, 26.11, 25.95, 22.86, 22.83, 21.49, 20.91, 19.46, 18.38, 18.13, 14.27, 14.23, 14.08, 11.97, 9.78. MS (MALDI-pos, DCTB): *m/z* (%): 2455.3 (32) [M+DCTB]$^+$; 2205.2 (100) [M]$^+$.

14a

To **13a** (78.5 mg, 33.8 µmol) in THF (10 mL), tetrabutylammonium fluoride (TBAF, 1 M in THF, 0.1 mL, 100 µmol) was added at rt, and the reaction mixture was stirred for 3 h. The reaction mixture was diluted with water and diethyl ether. The aqueous layer was extracted with diethyl ether, and the combined organic layers were washed with brine and dried with Na_2SO_4, before the solvent was evaporated under reduced pressure. Purification of the crude products by column chromatography using silica gel and CH/DCM 3:1 as eluent (R_f = 0.38) yielded **14a** (59.7 mg, 30.5 µmol, 90%) as yellow solid. Chemical formula: $C_{128}H_{162}O_8S_4$. Molecular weight: 1956.94 g/mol. ^{1}H NMR (400 MHz, C*D*Cl$_3$, RT): δ [ppm] = 7.75 (s, 2H), 7.75 (s, 2H), 7.46 (d, ^3J = 7.9 Hz, 2H), 7.44 (d, ^3J = 7.9 Hz, 2H), 7.10 (dd, ^3J = 7.8 Hz, ^4J = 1.3 Hz, 2H), 7.06 – 7.03 (m, 4H), 4.27 (t, ^3J = 6.5 Hz, 8H), 4.11 – 4.04 (m, 8H), 3.37

(s, 2H), 1.94 – 1.79 (m, 16H), 1.67 – 1.46 (m, 16H), 1.41 – 1.20 (m, 72H), 1.02 (t, ^3J = 7.3 Hz, 12H), 0.93 – 0.83 (m, 12H). ^{13}C NMR (101 MHz, C*D*Cl$_3$, RT): δ [ppm] = 160.82, 160.09, 142.90, 142.87, 135.06, 135.01, 134.34, 134.11, 130.65, 127.23, 127.16, 124.46, 124.19, 123.81, 123.69, 122.85, 122.80, 114.63, 112.61, 112.58, 95.11, 94.98, 85.39, 84.76, 82.96, 80.01, 79.87, 79.42, 73.70, 69.23, 69.10, 32.62, 32.09, 32.07, 29.89, 29.84, 29.82, 29.80, 29.73, 29.71, 29.54, 29.49, 29.16, 29.14, 26.10, 26.07, 22.85, 19.45, 14.28, 14.08. MS (MALDI-pos, DCTB): *m/z* (%): 2205.2 (12) [M+DCTB]$^+$; 1955.0 (100) [M]$^+$.

14b

To **13b** (73.8 mg, 33.4 µmol) in THF (10 mL), tetrabutylammonium fluoride (TBAF, 1 M in THF, 0.1 mL, 100 µmol) was added at rt, and the reaction mixture was stirred for 3 h. The reaction mixture was diluted with water and diethyl ether. The aqueous layer was extracted with diethyl ether, and the combined organic layers were washed with brine and dried with Na_2SO_4, before the solvent was evaporated under reduced pressure. Purification of the crude products by column chromatography using silica gel and CH/DCM 3:1 as eluent (R_f = 0.38) yielded **14b** (51.6 mg, 28.0 µmol, 84%) as yellow solid. Chemical formula: $C_{120}H_{146}O_8S_4$. Molecular weight: 1844.72 g/mol. ^{1}H NMR (400 MHz, C*D*Cl$_3$, RT): δ [ppm] = 7.75 (s, 2H), 7.75 (s, 2H), 7.46 (d, ^3J = 7.9 Hz, 2H), 7.44 (d, ^3J = 8.0 Hz, 2H), 7.10 (dd, ^3J = 7.8 Hz, ^4J = 1.4 Hz, 4H), 7.07 – 7.02 (m, 4H), 4.27 (t, ^3J = 6.5 Hz, 8H), 4.12 – 4.04 (m, 8H), 3.37 (s, 2H), 1.95 – 1.77 (m, 16H), 1.67 – 1.47 (m, 16H), 1.44 – 1.21 (m, 56H), 1.02 (t, ^3J = 7.3 Hz, 12H), 0.97 – 0.88 (m, 6H), 0.87 (t, ^3J = 6.7 Hz, 6H). ^{13}C NMR (101 MHz, C*D*Cl$_3$, RT): δ [ppm] = 160.83, 160.09, 142.90, 142.88, 135.07, 135.02, 134.35, 134.12, 130.66, 127.23, 127.16, 124.46, 124.20, 123.81, 123.70, 122.86, 122.81, 114.65, 114.63, 112.62, 112.59, 95.11, 94.98, 85.38, 84.76, 82.96, 80.01, 79.86, 79.42, 73.71, 69.24, 69.10, 32.62, 32.09, 31.66, 29.89, 29.84, 29.82, 29.80, 29.54, 29.14, 29.12, 27.07, 26.10, 25.75, 22.85, 22.74, 19.45, 14.27, 14.17, 14.08. MS (MALDI-pos, DCTB): *m/z* (%): 2343.2 (9) [M+2DCTB]$^+$; 2093.0 (27) [M+DCTB]$^+$; 1842.9 (100) [M]$^+$.

1a

Under an argon atmosphere, a catalyst mixture containing $Pd(PPh_3)_2Cl_2$ (6.42 mg, 9.15 µmol), CuI (4.65 mg, 24.4 µmol), I_2 (19.4 mg, 76.3 µmol) in dry THF (10 mL) and dry diisopropylamine (iPr_2NH, 20 mL) was heated to 50 °C, and **14a** (59.7 mg, 30.5 µmol) in dry THF (18 mL) was added over 48 h. The reaction mixture was stirred for another 24 h at 55 °C and diluted with water and DCM. The aqueous layer was extracted with DCM, and the combined organic layers were washed with water, aqueous HCl (10%), water, and brine, and dried over Na_2SO_4, before the solvent was removed under reduced pressure. The catalyst mixture was removed by purification of the crude product by column chromatography (filtration column) using silica gel and CH/DCM 2:1 as eluent. Subsequently, purification of the crude product by recycling gel permeation chromatography (recGPC) yielded **1a** (16.2 mg, 4.14 µmol, 27%) as yellow solid. Chemical formula: $C_{256}H_{320}O_{16}S_8$. Molecular weight: 3909.84 g/mol. ^{1}H NMR (500 MHz, $CDCl_3$, RT): δ [ppm] = 7.67 (s, 8H), 7.43 (d, ^3J = 7.7 Hz, 8H), 7.05 – 7.01 (m, 8H), 6.96 (s, 8H), 4.28 (t, ^3J = 6.3 Hz, 16H), 4.08 (t, ^3J = 6.7 Hz, 16H), 1.95 – 1.80 (m, 32H), 1.66 – 1.51 (m, 32H), 1.46 – 1.23 (m, 144H), 1.04 (t, ^3J = 7.4 Hz, 24H), 0.89 (t, ^3J = 6.9 Hz, 24H). ^{13}C NMR (126 MHz, $CDCl_3$, RT): δ[ppm] = 160.66, 142.93, 134.80, 134.47, 130.57, 127.91, 124.36, 123.88, 122.66, 114.47, 112.56, 94.97, 85.38, 80.12, 79.61, 73.64, 69.18, 32.70, 32.11, 32.10, 29.93, 29.91, 29.88, 29.85, 29.84, 29.82, 29.61, 29.57, 29.55, 29.23, 26.15, 22.89, 22.86, 19.49, 14.30, 14.28, 14.13. MS (MALDI-pos, DCTB): *m/z* (%): 4410.7 (7) $[M+2DCTB]^+$; 4160.3 (47) $[M+DCTB]^+$; 3909.8 (100) $[M]^+$. GPC (PS-calibration) $M_p = 5.9 \times 10^3$ g/mol.

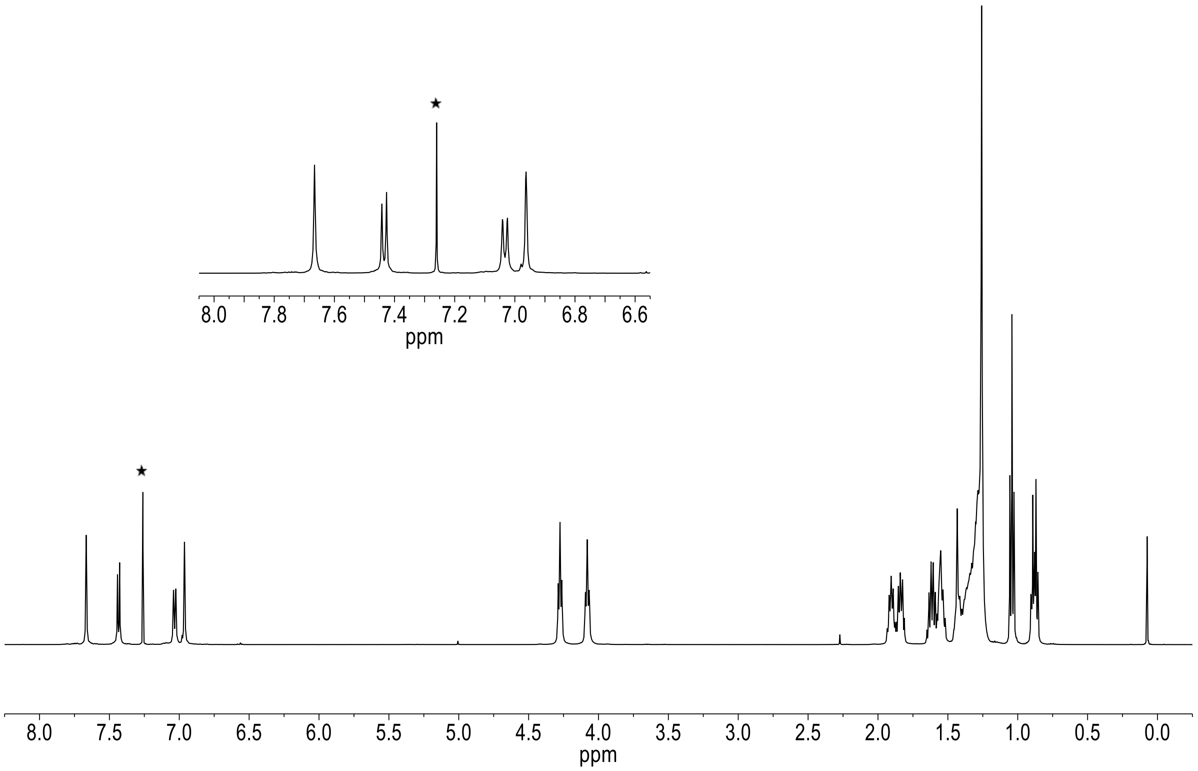

Figure S11: ^{1}H NMR of **1a** in $CDCl_3$ (*).

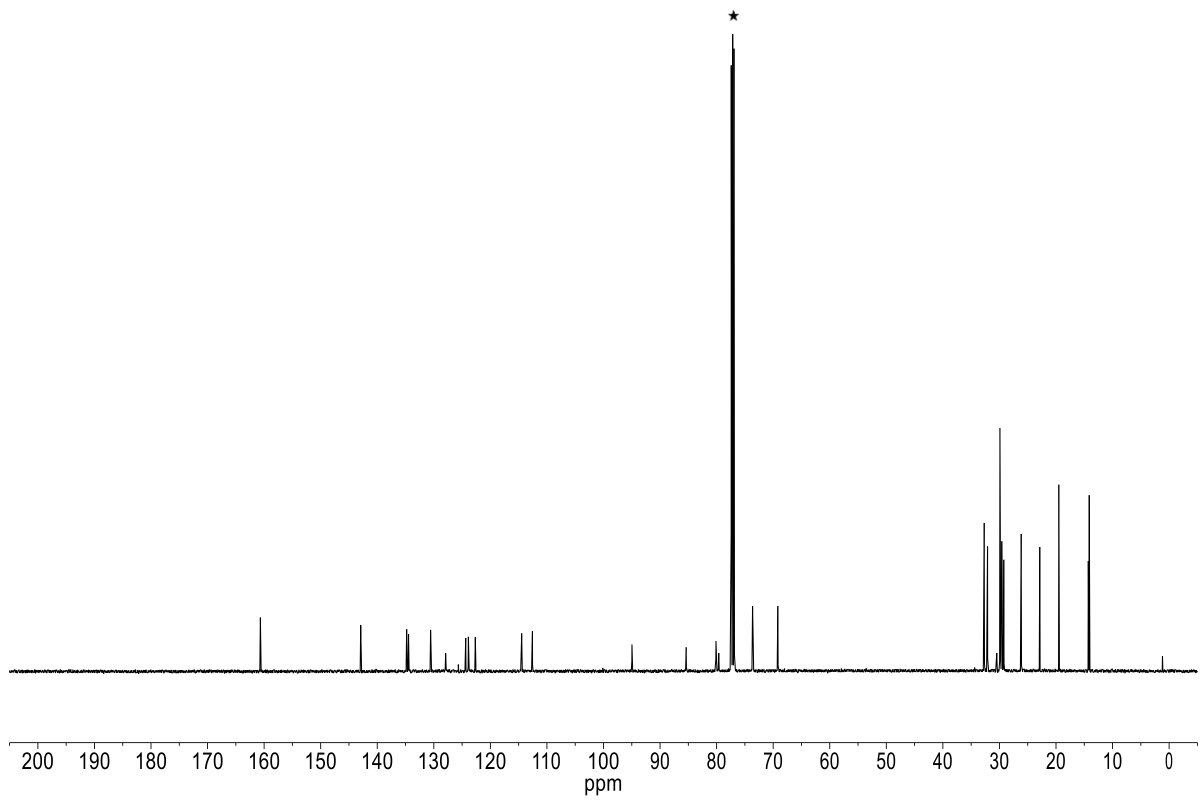

Figure S12: ^{13}C NMR of **1a** in $CDCl_3$ (*).

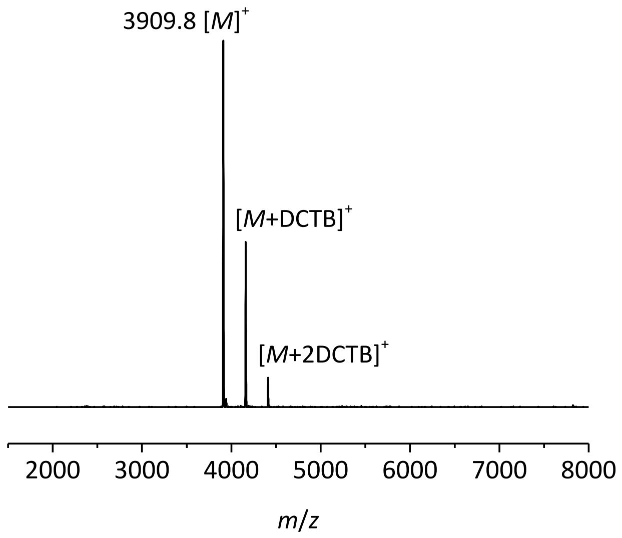

Figure S13: MALDI-TOF-MS of **1a**.

1b

Under an argon atmosphere, a catalyst mixture containing $Pd(PPh_3)_2Cl_2$ (5.89 mg, 8.39 µmol), CuI (4.57 mg, 24.0 µmol), I_2 (19.0 mg, 75.0 µmol) in dry THF (15 mL) and dry diisopropylamine (iPr_2NH, 15 mL) was heated to 50 °C, and **14b** (51.6 mg, 30.0 µmol) in dry THF (17 mL) was added over 48 h. The reaction mixture was stirred for another 24 h at 55 °C, and diluted with water and DCM. The aqueous layer was extracted with DCM, and the combined organic layers were washed with water, aqueous HCl (10%), water, and brine, and dried over Na_2SO_4, before the solvent was removed under reduced pressure. The catalyst mixture was removed by purification of the crude product by column chromatography (filtration column) using silica gel and CH/DCM 2:1 as eluent. Subsequently, purification of the crude product by recycling gel permeation chromatography (recGPC) yielded **1b** (7.4 mg, 2.01 µmol, 13%) as yellow solid. Chemical formula: $C_{240}H_{288}O_{16}S_8$. Molecular weight: 3685.41 g/mol. ^{1}H NMR (500 MHz, C*D*Cl$_3$, RT): δ [ppm] = 7.70 (s, 4H), 7.69 (s, 4H), 7.44 (d, ^3J = 7.7 Hz, 8H), 7.07 – 7.02 (m, 8H), 6.98 (s, 8H), 4.27 (t, ^3J = 6.4 Hz, 16H), 4.11 – 4.06 (m, 16H), 1.94 – 1.80 (m, 32H), 1.65 – 1.51 (m, 32H), 1.46 – 1.21 (m, 112H), 1.04 (t, ^3J = 7.4 Hz, 24H), 0.98 – 0.94 (m, 12H), 0.87 (t, ^3J = 6.8 Hz, 12H). ^{13}C NMR (126 MHz, C*D*Cl$_3$, RT): δ [ppm] = 160.68, 142.95, 134.84, 134.45, 130.57, 127.95, 124.39, 123.87, 122.68, 114.49, 112.58, 94.97, 85.43, 80.10, 79.60, 73.67, 69.19, 32.68, 32.10, 31.72, 29.92, 29.91, 29.86, 29.85, 29.60, 29.55, 29.21, 29.14, 26.15, 25.79, 22.86, 22.79, 19.49, 14.28, 14.24, 14.12. MS (MALDI-pos, DCTB): *m/z* (%): 4186.1 (17) [*M*+2DCTB]$^+$; 3935.9 (75) [*M*+DCTB]$^+$; 3685.4 (100) [*M*]$^+$. GPC (PS-calibration) $M_p = 5.6 \times 10^3$ g/mol.

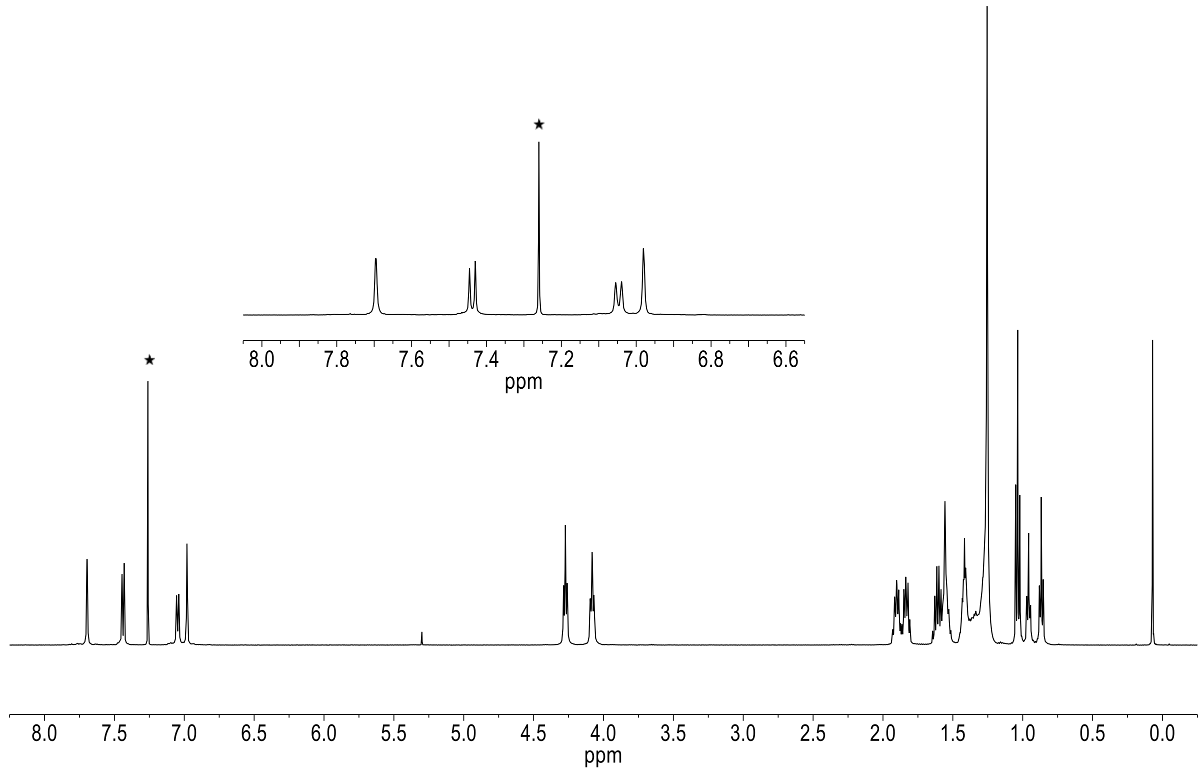

Figure S14: ^{1}H NMR of **1b** in $CDCl_3$ (*).

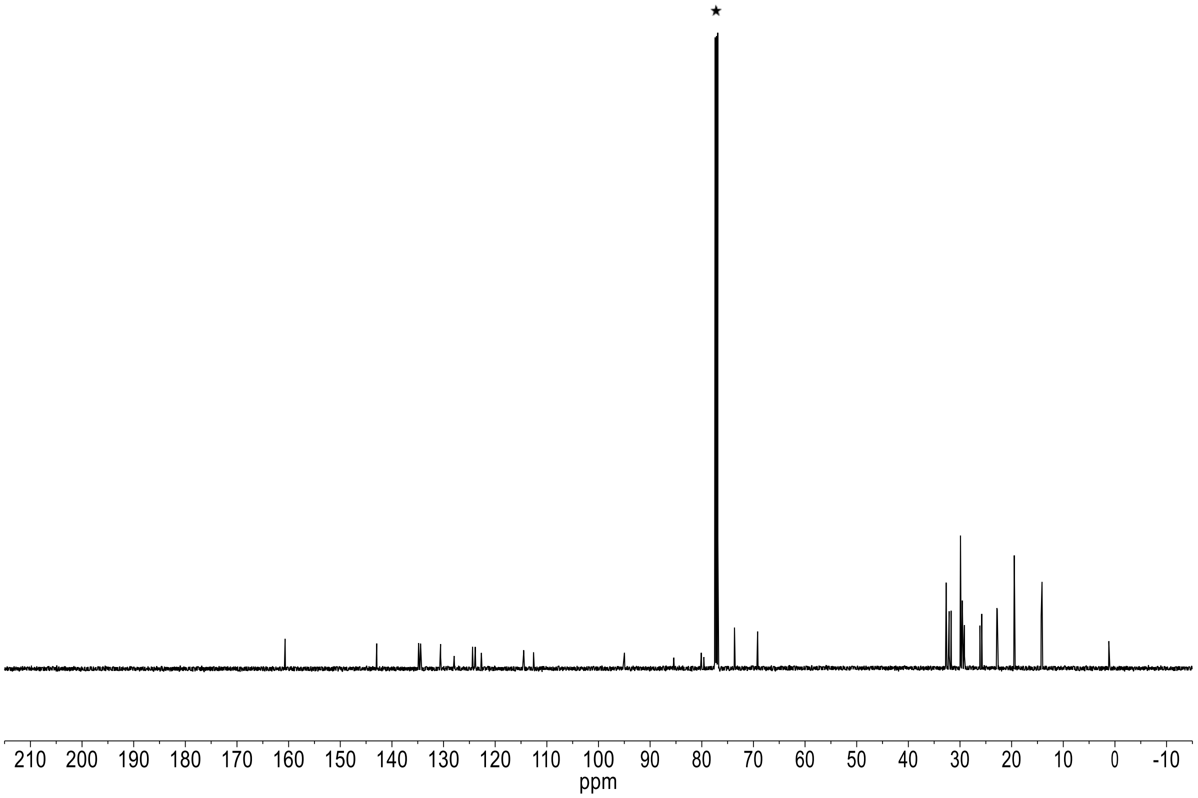

Figure S15: ^{13}C NMR of **1b** in $CDCl_3$ (*).

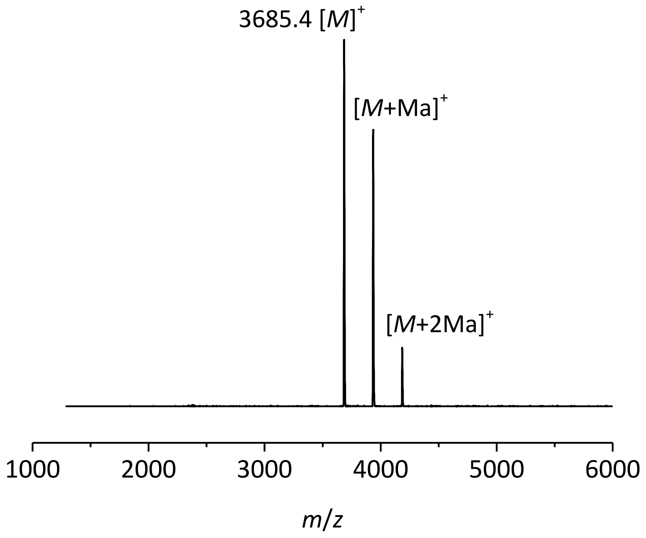

Figure S16: MALDI-TOF-MS of **1b**.

4. References

1. S.-S. Jester, E. Sigmund, S. Höger *J. Am. Chem. Soc.* **2011**, *133*, 11062-11065.
2. S. Höger, K. Bonrad, G. Schäfer, V. Enkelmann *Z. Naturforsch. B*, **1998**, *53*, 960–964.
3. S. Höger, K. Bonrad *J. Org. Chem.* **2000**, *65*, 2243–2245.
4. G. Gaefke, S. Höger *Synthesis* **2008**, 2155–2157.
5. P. Wilhelm, J. Vogelsang, G. Poluektov, N. Schönfelder, T. J. Keller, S.-S. Jester, S. Höger, J. M. Lupton *Angew. Chem.* **2017**, *129*, 1254–1258; *Angew. Chem. Int. Ed.* **2017**, *56*, 1234–1238.
